# Evoked responses to rhythmic visual stimulation vary across sources of intrinsic alpha activity in humans

**DOI:** 10.1038/s41598-022-09922-2

**Published:** 2022-04-08

**Authors:** R. Nuttall, C. Jäger, J. Zimmermann, M. E. Archila-Melendez, C. Preibisch, P. Taylor, P. Sauseng, A. Wohlschläger, C. Sorg, J. Dowsett

**Affiliations:** 1 School of Medicine, TUM-Neuroimaging Center, Technical University of Munich, Munich, Germany; 2 Department of Neuroradiology, School of Medicine, Technical University of Munich, Munich, Germany; 3 Department of Neurology, School of Medicine, Ludwig Maximilian University, Munich, Germany; 4 School of Medicine, German Center for Vertigo and Balance Disorders, Ludwig Maximilian University, Munich, Germany; 5 Munich Center for Neurosciences – Brain and Mind, Ludwig Maximilian University, Munich, Germany; 6 Department of Anesthesiology and Intensive Care, School of Medicine, Technical University of Munich, Munich, Germany; 7 Department of Psychology, Ludwig Maximilian University, Munich, Germany

**Keywords:** Neuroscience, Systems biology

## Abstract

Rhythmic flickering visual stimulation produces steady-state visually evoked potentials (SSVEPs) in electroencephalogram (EEG) recordings. Based on electrode-level analyses, two dichotomous models of the underpinning mechanisms leading to SSVEP generation have been proposed: entrainment or superposition, i.e., phase-alignment or independence of endogenous brain oscillations from flicker-induced oscillations, respectively. Electrode-level analyses, however, represent an averaged view of underlying ‘source-level’ activity, at which variability in SSVEPs may lie, possibly suggesting the co-existence of multiple mechanisms. To probe this idea, we investigated the variability of SSVEPs derived from the sources underpinning scalp EEG responses during presentation of a flickering radial checkerboard. Flicker was presented between 6 and 12 Hz in 1 Hz steps, and at individual alpha frequency (IAF i.e., the dominant frequency of endogenous alpha oscillatory activity). We tested whether sources of endogenous alpha activity could be dissociated according to evoked responses to different flicker frequencies relative to IAF. Occipitoparietal sources were identified by temporal independent component analysis, maximal resting-state alpha power at IAF and source localisation. The pattern of SSVEPs to rhythmic flicker relative to IAF was estimated by correlation coefficients, describing the correlation between the peak-to-peak amplitude of the SSVEP and the absolute distance of the flicker frequency from IAF across flicker conditions. We observed extreme variability in correlation coefficients across sources, ranging from −0.84 to 0.93, with sources showing largely different coefficients co-existing within subjects. This result demonstrates variation in evoked responses to flicker across sources of endogenous alpha oscillatory activity. Data support the idea of multiple SSVEP mechanisms.

## Introduction

Rhythmic flickering light produces steady state visually evoked potentials (SSVEPs) measured via electroencephalography (EEG)^1,2^. SSVEPs have been extensively utilised as a versatile tool for investigating higher-order cognitive processes such as attention via an approach called ‘frequency tagging’ during EEG and magnetoelectroencephalography (MEG) measurements, which enables the measurement of stimuli-specific neural responses^3^. Recent studies have used SSVEPs to investigate functional specialisation during tasks utilising selective spatial attention^4^ and object-based attentional paradigms, utilising MEG with additional structural magnetic resonance imaging to locate functional regions/networks involved in the specific processing of various parts of the face^5^ and houses^6^. Interestingly, a recent MEG study^7^ utilised quasi-rhythmic visual stimulation to investigate the cortical tracking of dynamic stimuli, across and within multiple frequency bands. They reported widespread cortical tracking via source localisation of the stimuli, regardless of stimuli frequency, which was enhanced by attention.

SSVEPs have also proven useful in the study of the function of oscillatory activity in the human brain via flicker stimulation at a rate that matches the intrinsic resonance frequency of the oscillatory system^2,8–17^. Alpha oscillations, for example, have been shown to play a causal^18^ role in attentional allocation based on changes in amplitude at ipsilateral versus contralateral visual cortices to target versus distractor stimuli; the so-called ‘gating by inhibition’ mechanism^19–23^.

SSVEP amplitudes to rhythmic flickering light within the alpha range have been shown to be maximal near or at the dominant frequency of endogenous alpha activity for a given individual during resting-state eyes closed, i.e., individual alpha frequency (IAF)^24,25^, suggesting that the flicker interacts with the ongoing alpha oscillator underpinning the IAF, potentially bringing the phase of this oscillator gradually into alignment with the flicker, leading to synchronisation between the two, i.e., ‘entraining’^8,12,14^. In support of this theory of entrainment, a recent study found that the presentation of stimuli at different phases of a 10 Hz rhythmic flicker affects target detection performance^16^, effects that can be predicted based on the distance of 10 Hz from IAF^9^.

Such an entrainment model would assume that the entrained oscillation is functionally identical to the endogenous oscillation^26^. However, a previous study showed that the driven oscillator is not functionally identical to the endogenous oscillation^11^; while an endogenous oscillator shows reductions in amplitude at recording sites contralateral to an attended stimulus^27^, SSVEP amplitude reliably increases^11^ and remains unrelated to the suppression of distractor processing^28^. Furthermore, recent findings^29,30^ demonstrated a dissociation between SSVEPs and ongoing alpha oscillations in terms of their attentional modulation during a spatial cueing task and a fear-conditioning paradigm, respectively. These findings support another mechanistic model of the SSVEP—one of superposition^31^. This model assumes no interaction between the ongoing oscillator and rhythmic stimulation; the SSVEPs and the endogenous oscillators remain independent and SSVEPs represent a series of event-related potentials that superimpose to a nearly periodic response.

Distinguishing between these two mechanistic models of the SSVEP is challenging and may require dissociating between the scalp and source level of analysis^32^. Averaged scalp-level EEG oscillations are underpinned by multiple distinct alpha sources that show functional and interindividual variability^33,34^. This variability, present at the source-level, is lost at the scalp: in a recent paper, alpha oscillators at the scalp-level showed a decrease in peak frequency and an increase in power across the resting periods of two experimental sessions. This was different at the source-level, with sources identified through a combined temporal-spatial analysis approach using temporal independent component analysis (tICA) and source localisation; some sources showed only a change in power, others only a change in IAF, and others a mixture of the two^35^. Distinct alpha sources may, therefore, show dissociable profiles, raising the possibility that different sources might show different responses to rhythmic flicker around IAF. Such variability in evoked responses across sources of endogenous alpha oscillatory activity could offer first insight into the possibility of varying interactions or contributions of intrinsic alpha oscillators with/towards the evoked responses at the source-level.

**Figure 1:**
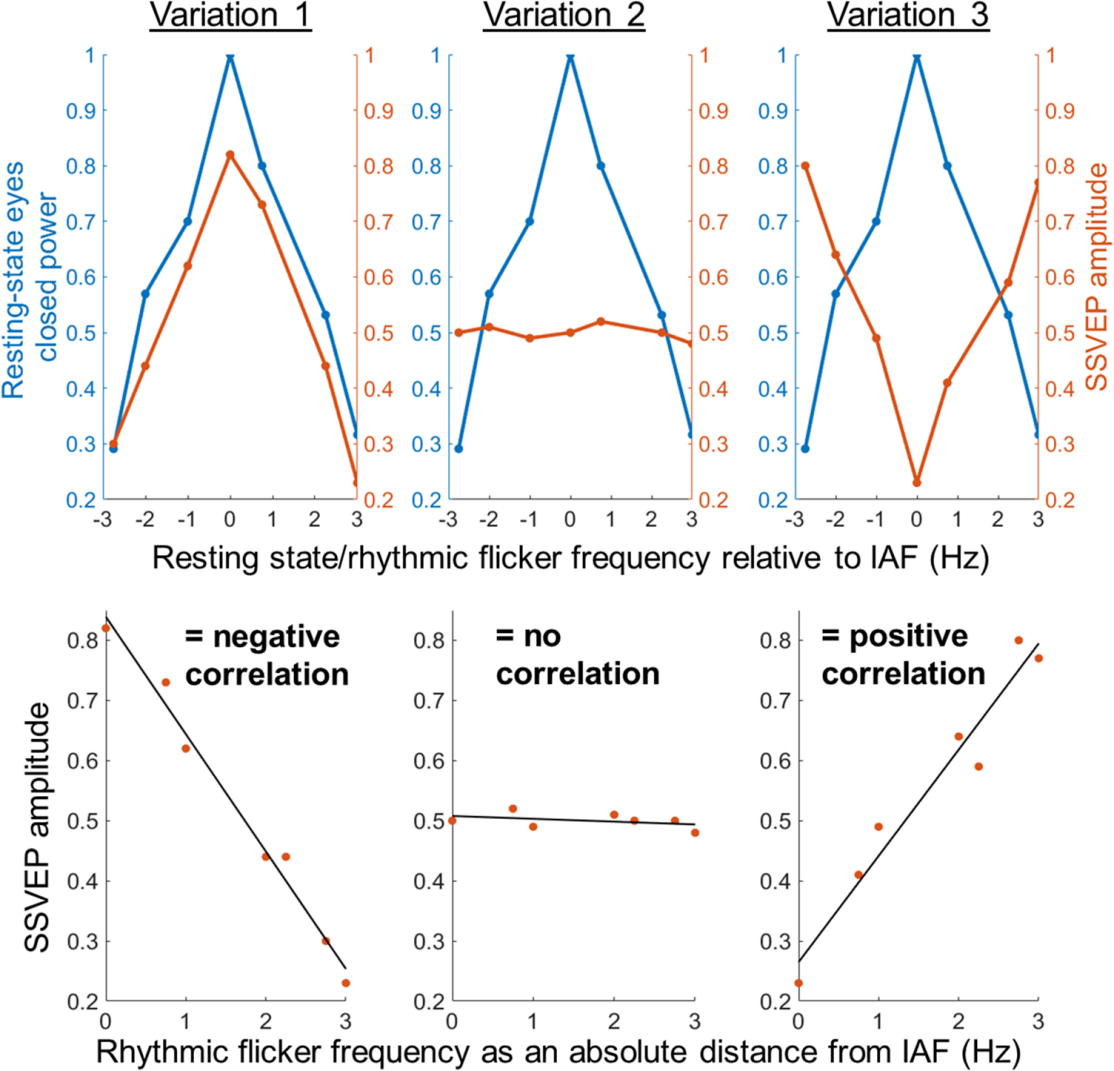
Hypothesis: variability of SSVEP amplitude relative to individual alpha frequency (IAF) at the source-level. Schematic illustration based on conceptual data of the hypothesised variability in SSVEP amplitude during rhythmic flicker stimulation relative to the IAF. First row, three possible variations in SSVEP amplitude profiles to rhythmic flicker relative to IAF are shown. All three oscillators show a peak in power at the IAF during resting state eyes closed (shown in blue) but heterogeneity in SSVEP amplitude to rhythmic flicker relative to IAF (shown in orange). Column 1, Variation 1 shows a maximal SSVEP amplitude at the IAF that decreases with stimulation frequencies further from the IAF, which leads to a negative correlation coefficient between the rhythmic flicker frequency as an absolute distance from IAF and the SSVEP amplitude. Column 2, Variation 2 shows no change in SSVEP amplitude as a function of the rhythmic flicker frequency’s absolute distance from IAF, leading to a near-zero correlation coefficient between the SSVEP amplitude and the absolute distance of the rhythmic flicker frequency from IAF. Column 3, Variation 3 shows a minimal SSVEP amplitude at the IAF that increases with stimulation frequencies further from the IAF, which leads to a positive correlation coefficient between the rhythmic flicker frequency as an absolute distance from IAF and the SSVEP amplitude.

We hypothesised that there would be significant variability across alpha sources, derived via tICA, in terms of their pattern of SSVEP amplitude as a function of the flicker frequency relative to IAF. We hypothesised three possible variations. Firstly, we expected a variation whereby a maximal SSVEP amplitude is found in response to rhythmic flicker near IAF (Fig. 1, ‘Variation 1’), as reported in previous literature^25^ and predicted under the entrainment model. Secondly, we expected a pattern of no interaction with the rhythmic flicker, i.e., no change in the SSVEP amplitude, regardless of flicker frequency distance from IAF (Fig. 1, ‘Variation 2’). This pattern could be expected, for example, under the superposition model. A final possible variation would be a minimal evoked response to IAF flicker as compared to other flicker frequencies (Fig. 1, ‘Variation 3’). This would indicate a frequency-specific interaction with the endogenous alpha oscillator, albeit one which cannot be explained by the traditional entrainment model. Thus, significant variability in the pattern of the evoked response to rhythmic flicker around the IAF at sources of endogenous alpha oscillatory activity could indicate variability in the way alpha generators deal with visual flicker relative to IAF.

## Materials and methods

### Participants and experimental design

Sixteen participants with normal/corrected vision were recruited (mean age 26 years with a standard deviation of 3 years, 10 female) and provided informed consent. The study was approved by the local ethics committee of Technical University Munich, Germany and the methods employed were in accordance with all relevant guidelines and regulations. Participants had no current, or history of, neurological or psychiatric disorders and did not take psychotropic medication. Structural brain abnormalities were excluded by conventional clinical MRI assessment (T1, T2, FLAIR).

The experiment was conducted in a dark room and participants were asked to lie down supine on a flat surface of height 47 cm and fixate on a projector screen (78 × 98 cm) placed 150 cm behind them through a mirror (71° visual angle) in order to allow for maximal comparability between the EEG data. Lying position was chosen as subjects performed a subsequent fMRI experiment of visual flicker stimulation in the same position to maximally exploit experimental resources. The current study focuses only on the EEG dataset.

The experiment was presented using Presentation software (Neurobehavioral Systems, http://www.neurobs.com) via a projector (In Focus LP530), placed 160 cm away from the projector screen and consisted of alternating periods of baseline and visual flicker stimulation. During baseline periods, a central red fixation cross on a black screen was presented for 10 s. Baseline periods were used to allow subjects to rest their eyes in between stimulation periods. Following each baseline period was a flicker stimulation period of 20 s. During stimulation periods, the visual stimulus consisted of a low intensity (luminance contrast ratio = 167 L/cd/m^2^) radial checkerboard stimulus with a central red fixation cross. Subjects were instructed to simply fixate on the cross and try to keep movement to a minimum. Subject eye positions were not measured. To provide the visual flicker, light was projected through a custom-built window made of LCD glass, which can be darkened when a voltage is applied across it. Hence, the flicker was created via a change in luminance. The LCD glass was controlled by a microcontroller (Arduino Uno, Scarmagno, Italy) which allowed the image to be made to flicker at any chosen frequency. The flicker was created with a 50% duty cycle, e.g., if the flicker frequency was 10 Hz the glass was darkened for 50 ms and transparent for 50 ms, summing to the 100 ms period. This provided constant average luminance across all flicker frequencies. The voltage pulse train controlling the LCD glass was split and additionally used as a trigger input, recorded with the EEG. This gave a trigger time-locked to the darkening of the LCD glass for each flicker, which was used for later segmentation of the data^36^. Each experimental block consisted of eight randomised flicker frequencies: 6–12 Hz in 1 Hz steps as well as at the IAF. IAF was measured during an initial supine, eyes closed, resting-state period of 60 s, from which the peak frequency in the alpha range (8–12 Hz) was found via taking the mean FFT power spectrum across 4-s baseline-corrected Hanning-windowed segments (resolution 0.25 Hz) in occipital and parietal electrodes (O1, O2, Oz, P1, P2, and Pz).

The eight flicker frequency conditions (i.e., baseline with fixation cross and flicker stimulation) together constituted an experimental block. Each experimental block was repeated three times, with breaks in-between for the subject to rest. Three subjects’ data were excluded from further analysis as one subject could not complete the testing, one had strong movement artifacts in their data, and one subject did not have any occipitoparietal sources of endogenous alpha frequency meeting inclusion criteria (see below 2.3.2), leaving 13 subjects’ data, consisting of three blocks, each with eight frequencies of rhythmic flicker stimulation for 20 s each.

### Data acquisition: EEG recording

EEG was recorded using a 64 channel (including an electrocardiogram (ECG) channel) MR-compatible EEG 10–10 system (BrainAmp MR plus, Brain Products, Munich Germany) with an online low pass filter of 250 Hz and a sampling rate of 1000 Hz. EEG was amplified in the range of ± 3.28 mV at a resolution of 0.1 μV/bit. Impedance was kept below 10kΩ. The online reference was located at FCz and the ground at AFz.

### Data analysis and outcome measure: correlation coefficient of the relationship between SSVEP-peak-to-peak amplitude and absolute distance of rhythmic flicker frequency from IAF

**Figure 2:**
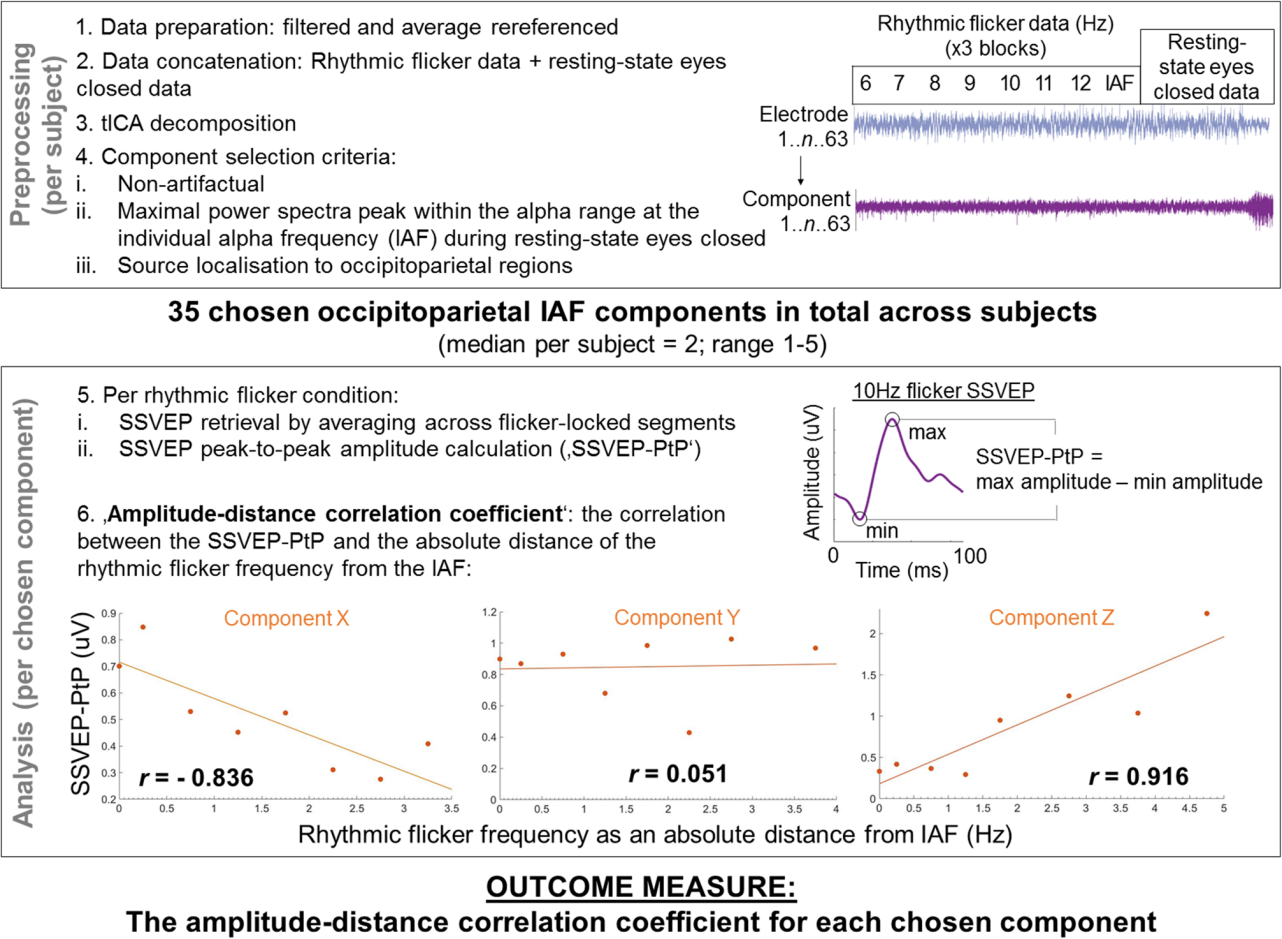
Analysis pipeline. Filtered and average re-referenced continuous data were epoched into rhythmic flicker 20 s stimulation trials and combined across blocks along with the 60 s resting-state eyes closed data and entered into a temporal ICA to derive 63 independent components across rhythmic stimulation trials and resting-state eyes closed per subject (*n* = 14). For each subject, independent components with a maximal peak in the alpha range at the individual alpha frequency (IAF), above 1/f aperiodic signal, during resting state eyes closed and source localised to occipitoparietal regions were chosen for further analysis as our alpha oscillators, resulting in 35 distinct alpha oscillatory components across all subjects (range 1–5 alpha components per subject, median per subject = 2). For each of these 35 chosen components, the peak-to-peak amplitude of the SSVEP (‘SSVEP-PtP’) per rhythmic flicker frequency condition was retrieved and correlated with the absolute distance of the rhythmic flicker frequency from IAF, giving one amplitude-distance correlation coefficient of the relationship between the SSVEP-PtP and the absolute distance of the rhythmic flicker frequency from IAF per chosen component as our outcome measure. Three different patterns based on real data are shown (example components X, Y, Z), representing the hypothesised variability in the SSVEP-PtP across occipitoparietal alpha oscillators as an absolute distance of rhythmic flicker frequency from IAF.

Data analysis pipeline is depicted in Fig. 2 and described in detail in the following section.

#### Preprocessing

Preprocessing and analyses used MATLAB (Mathworks, v2016b), EEGLAB^37^ (v2019.0, https://sccn.ucsd.edu/eeglab/index.php) and Python^38^ (v3.6.4, packages ‘NumPy’, ‘SciPy’). The continuous data across the course of the experiment was average re-referenced, following which a zero-phase FIR filter was applied (band pass: 0.1–40 Hz, −6 dB/octave, roll-off: [0.05 Hz 40.05 Hz]).

#### Source definition

**Figure 3:**
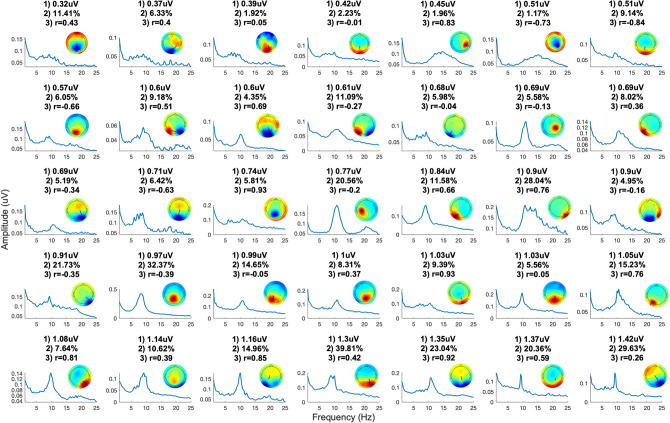
Chosen component characteristics. Power spectra and weights are plotted across all chosen components. Each component is plotted with details of (1) resting state amplitude of the power spectral peak at the IAF; (2) percentage variance in the raw data accounted for by the component; (3) the amplitude-distance correlation coefficient. Components are ordered by increasing resting state amplitude at the IAF.

The 20 s of rhythmic flicker for each of the eight rhythmic flicker frequency conditions were concatenated across all three blocks and further concatenated with the 60 s of resting state eyes-closed data. This concatenated data was entered into a tICA using the extended ICA algorithm^39^ implemented in the EEGLAB toolbox, giving 63 temporally independent components (the ECG channel was excluded) spanning the stimulation blocks and the resting-state eyes closed period. Per subject, the independent components were entered into the ICLabel toolbox (Swartz Center for Computational Neuroscience, https://sccn.ucsd.edu/wiki/ICLabel) to identify components that were most likely to represent brain activity. These ‘non-noise’ components’ time-series were then segmented, taking only the 60 s resting-state eyes closed data, and entered into the FOOOF toolbox^40^ which allows for the identification of periodic power spectral peaks after 1/f correction, i.e., removal of the aperiodic activity. For each subject, components with a maximal peak above 1/f aperiodic power within the alpha range at the IAF were taken as alpha oscillators. Only those alpha oscillators with a maximal amplitude at the IAF during resting-state eyes closed were taken to ensure that the IAF measured at the scalp-level represents the endogenous frequency of each source-level component analysed. Source localisation of components using DIPFIT^41^ (Donders Center, University of Nijmegen) revealed the likely source origin of each component, using a three-shell boundary element model (BEM) of the MNI standard brain, with singular dipoles plotted on each subject’s normalised MPRAGE MRI scan (normalised via SPM12 (https://www.fil.ion.ucl.ac.uk/spm/software/spm12/), (0.75 mm^3^ isotropic resolution), on a Philips Ingenia 3 T scanner acquired within 21 days of the EEG acquisition. Any of our remaining components representing alpha oscillators were excluded if their source localisation estimated an area of highest probability outside occipital or parietal regions (labelled automatically in the DIPFIT output using the Desikan-Killiany atlas^42^, or if the residual variance of the overlap between each component’s scalp map and the projected scalp map of the equivalent dipole exceeded 15%. As mentioned above, one subject was excluded entirely after showing no components meeting these criteria. This resulted in 35 occipitoparietal alpha components across all remaining 13 subjects (range per subject 1–5, median per subject = 2, see Fig. 3 for component scalp topographies, power spectra, percentage variance accounted for by the component in the original data and the outcome variable, amplitude-distance correlation coefficient).

#### SSVEP-peak-to-peak amplitudes of a source

The time-series of each chosen component was segmented into the rhythmic flicker stimulation periods of 20 s for each of the three blocks. SSVEPs were derived from these source components by further segmenting each 20 s period into individual time-locked flicker segments and averaging across all flickers across blocks to give one SSVEP waveform per rhythmic flicker frequency condition. To characterise the amplitude of each SSVEP, the minimum amplitude value of the SSVEP was subtracted from the maximum amplitude value giving one value of *peak-to-peak amplitude of the SSVEP (i.e., SSVEP-PtP)* for each of the eight rhythmic flicker conditions. We used this peak-to-peak amplitude approach in order to avoid any further assumptions on our data. For example, the typical FFT approach assumes neural oscillatory activity to be sinusoidal^43^, which SSVEPs often do not fulfil.

#### Complementary analysis for SSVEP-PtP amplitude approach

In order to offer a complementary analysis to our results obtained using the SSVEP peak-to-peak amplitude, we performed an additional evoked FFT analysis. This allowed us to obtain the amplitude of the evoked response per rhythmic flicker condition via the more traditional approach. We also investigated the waveform shapes of our evoked responses by comparing the power at the fundamental frequency (i.e., flicker frequency) with the power at the first harmonic (see Supplementary Analysis S1 for detailed methodology). The statistical significance of the evoked responses themselves to flicker were also calculated in a supplementary analysis looking at the fundamental and second harmonic evoked amplitude to flicker across chosen components, expressed as z-scores (see Supplementary Analysis S4 and Figure S8).

#### Outcome measure amplitude-distance correlation coefficient

Each of the eight rhythmic flicker frequencies were transformed to reflect how far away the stimulation frequency was from the IAF in absolute terms (*flicker-IAF distance*), e.g., given an IAF of 9.25 Hz, the 6 Hz condition would be 3.25 Hz as an absolute distance from the IAF, and the 11 Hz condition would be 1.75 Hz. The Pearson’s correlation coefficient between the SSVEP-PtP and the flicker-IAF distance was taken as our outcome measure, the *amplitude-distance correlation coefficient*, for each of our 35 chosen occipitoparietal alpha oscillators. This value describes the pattern of how SSVEP amplitudes to rhythmic flicker stimulation vary as a function of flicker-IAF distance. A positive correlation coefficient would indicate a minimal SSVEP-PtP at the IAF, increasing as a function of flicker-IAF distance. A negative correlation coefficient would indicate a maximal SSVEP-PtP at the IAF that decreases as a function of flicker-IAF distance. A near-zero correlation coefficient would indicate no change in the SSVEP-PtP as a function of flicker-IAF distance. Note that an amplitude inversely proportional to frequency regardless of IAF (i.e., simply following the 1/f decay) would result in increasing SSVEP-PtP for rhythmic flicker frequencies less than the IAF and a decreasing SSVEP-PtP for rhythmic flicker frequencies greater than the IAF and would also result in a near-zero correlation.

### Statistical analysis

#### Hypothesis testing

We hypothesised that there would be significant variability in the amplitude-distance correlation coefficient across our occipitoparietal alpha oscillators. To address this hypothesis, we performed a permutation test with 1000 repetitions to sample the distribution of standard deviation values expected by chance. Specifically, per repetition, SSVEP-PtP values were shuffled across rhythmic flicker conditions, from which a new amplitude-distance correlation coefficient was derived for each of our 35 chosen components and a permuted standard deviation value retrieved, resulting in 1000 permuted standard deviation values of the amplitude-distance correlation coefficients across components. *P*-values were calculated from the normalised distance of our observed standard deviation from the distribution of the permuted standard deviation values, following the procedure previously described^44^.

#### Control analysis: sensitivity of analysis pipeline to outliers

A Pearson’s correlation of eight data points is highly sensitive to individual outliers. Permutation tests are robust against outliers because any outliers are included in the random reshuffling of the data. To quantify how susceptible our analysis pipeline was to outliers, simulated data was created to (1) model the change in the correlation coefficient that could be observable by outliers of different sizes, and (2) directly compare the results obtained with our analysis pipeline on data with trends driven by our hypothesis and data with trends driven by outliers (see Supplementary Analysis S2 for detailed methodology).

#### Explorative analysis

We performed a further exploratory analysis, in order to understand better which factors might be relevant for the variability of amplitude-distance correlation coefficients across occipitoparietal alpha oscillators. We focused on intrinsic amplitude at IAF of alpha oscillators at rest as a potential factor; we investigated the relationship between the amplitude-distance correlation coefficient and the intrinsic amplitude at the peak IAF during resting-state eyes closed across components.

This analysis was approached statistically in two ways. Firstly, a linear mixed-model analysis using the maximum-likelihood method was performed to account for the violation of non-independence due to multiple data points coming from any one subject. The intercept of the linear fitting was varied across subjects, effectively controlling for inter-subject variability in terms of their amplitude-distance correlation coefficients. Secondly, the components were split into three groups and three permutation tests were performed to test whether the observed mean amplitude-distance correlation coefficient for each group could be expected by chance. Using a similar approach to a previous study^45^, the 35 chosen components were split into three groups based on their intrinsic amplitude at resting-state eyes closed at the IAF as retrieved from the FOOOF output: low (amplitude < 0.62 uV), mid (amplitude between 0.62 and 0.972 uV) and high (amplitude > 0.972 uV). These groupings were chosen to keep the number of components and subject distribution per group as equal as possible (low: *n* components = 11 from 5 subjects; mid: *n* components = 12 from 6 subjects; high: *n* components = 12 from 7 subjects). A permutation test was performed on each group by shuffling the SSVEP-PtP values across the eight rhythmic conditions and acquiring a new permuted mean amplitude-distance correlation coefficient across all components within each group, permuted 1000 times. This gave us a distribution of 1000 permuted mean amplitude-distance correlation coefficients as expected by chance for each group of components. From these distributions, a *p*-value indicating the statistical significance of our observed mean amplitude-distance correlation coefficient for each group could be calculated and corrected for multiple comparisons via the Bonferroni correction.

## Results

**Figure 4:**
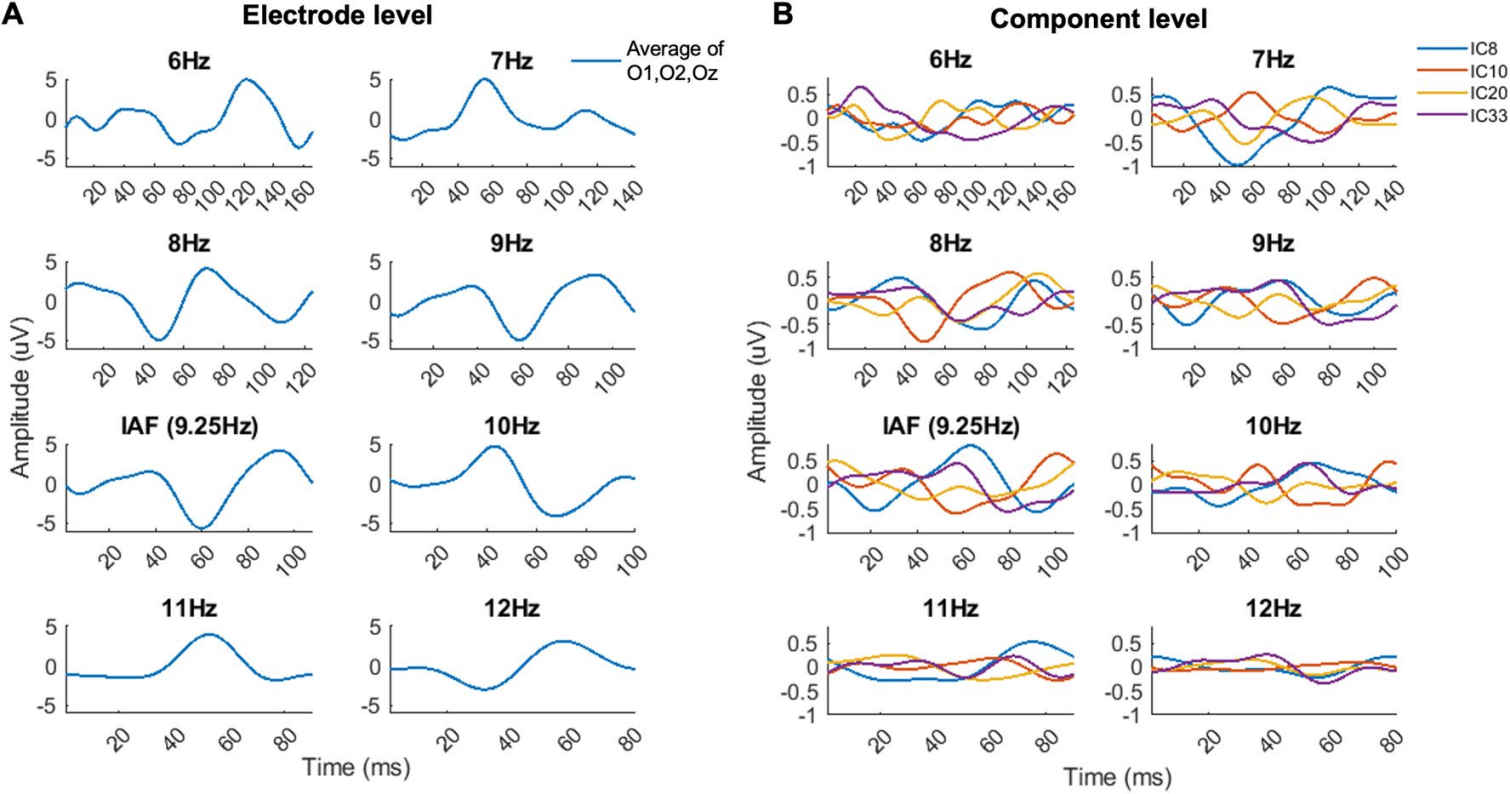
Electrode versus component level SSVEPs for one subject. (Panel **A**). Electrode level. The average SSVEP across electrodes O1, O2 and Oz are shown for each rhythmic flicker frequency condition. (Panel **B**). Component level. The SSVEP for each of the four components for this subject are shown for each of the rhythmic flicker frequency conditions.

**Figure 5:**
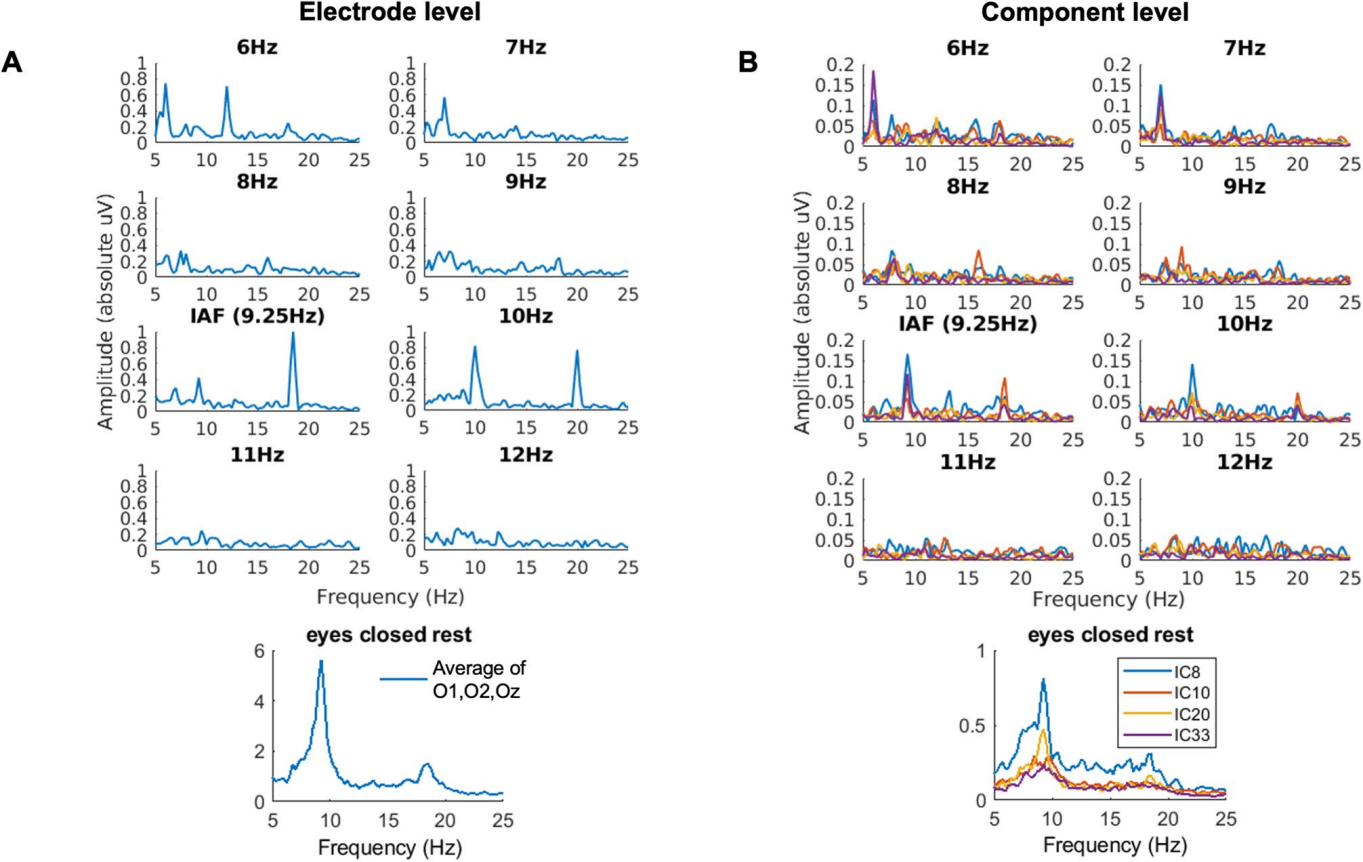
Electrode versus component level power spectra for one subject. (Panel **A**). Electrode level. The average FFT-derived power spectrum across electrodes O1, O2 and Oz are shown for each rhythmic flicker frequency condition and the eyes closed rest condition. (Panel **B**). Component level. The FFT-derived power spectra for each of the four components for this subject are shown for each of the rhythmic flicker frequency conditions and the eyes closed rest condition.

In order to test for the variability of SSVEPs formed from sources that underpin the IAF, we performed systematic rhythmic stimulation around IAF within the alpha frequency band. Example data for a single subject are presented in Fig. 4 and 5, showing the SSVEP waveform and the evoked power spectra, respectively, for each of the eight rhythmic flicker frequency conditions. Panel A shows results at the electrode level, taken as an average across electrodes O1, O2 and Oz, and Panel B shows results at the component level, for each of the four chosen alpha components for this subject. Note the variability in evoked waveform shape and power spectra for each component that is no longer seen at the electrode level.

**Figure 6:**
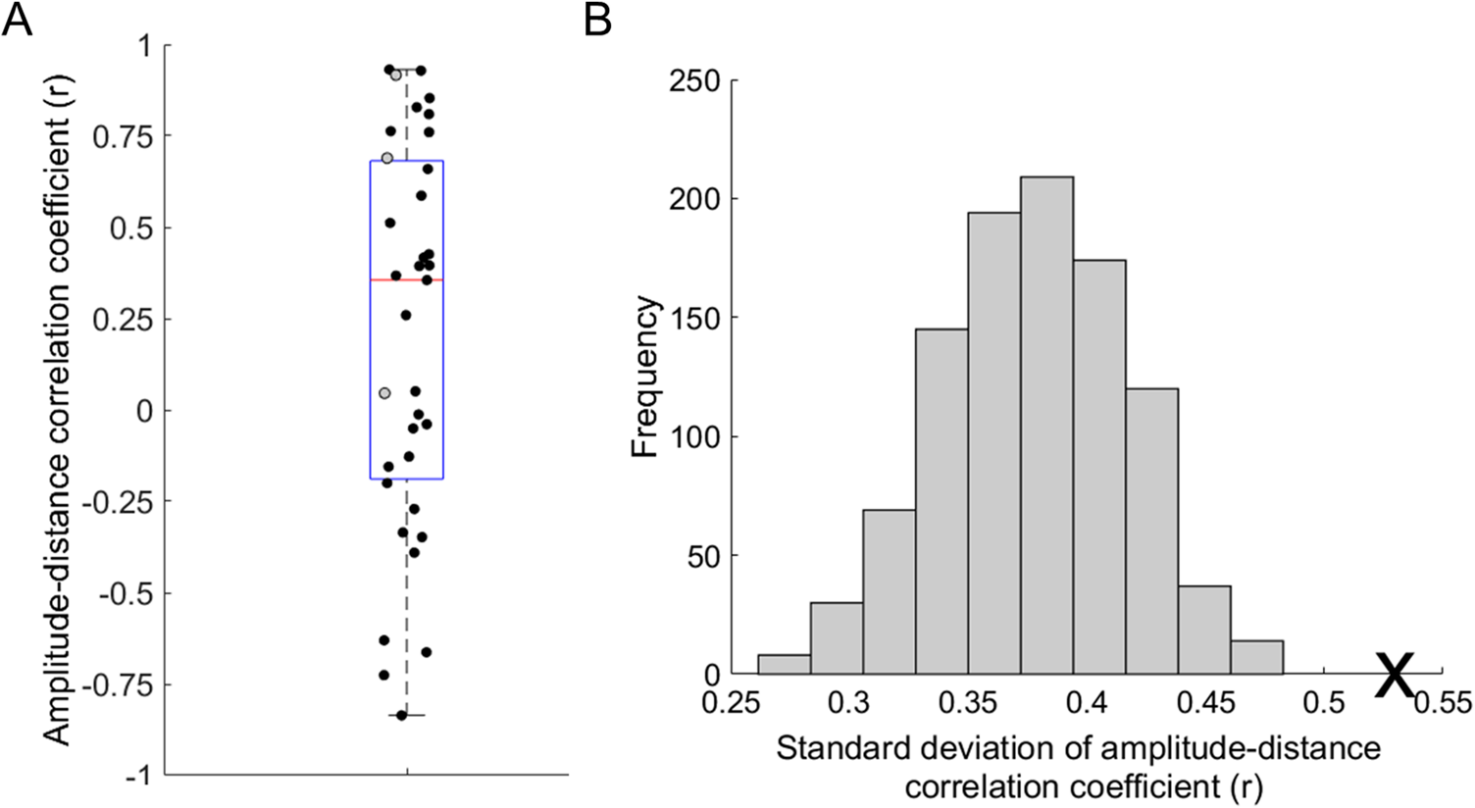
Variability in the visually evoked response of distinct occipitoparietal alpha oscillations to rhythmic flicker relative to IAF. (Panel **A**), Boxplot illustrating the significant variability in the amplitude-distance correlation coefficients across the 35 alpha oscillatory components, with the horizontal red line representing the median, and the top and bottom edges of the blue box representing the 75th and 25th percentiles, respectively. Three subjects showed only one source (plotted as grey circles), the remaining ten subjects showed multiple sources distributed across the three possible amplitude-distance correlation coefficient variations (plotted as black circles). (Panel **B**), A histogram, illustrating the distribution of randomly acquirable standard deviations in amplitude-distance correlation coefficients estimated from a permutation test across all 35 alpha oscillators, which showed that our observed standard deviation value of 0.527 (marked by a black cross) lies well outside of the range of standard deviation values likely to occur by chance (permuted mean standard deviation = 0.3773, shown in grey bars) retrieved from 1,000 permutations, *p* = 0.000056.

The range of SSVEPs of a given source are captured by our outcome measure of ‘amplitude-distance correlation coefficient’, which reflects the relationship between SSVEP amplitude and flicker-IAF distance. Positive correlation coefficients reflect sources whose SSVEP is minimal at IAF; negative coefficients indicate sources with a maximal SSVEP amplitude at IAF; and sources with a near-zero coefficient reflect a missing relationship between SSVEP amplitude and distance of stimulation frequency to IAF. Supplementary Figure S1 shows the SSVEP amplitude to rhythmic flicker relative to IAF at the electrode versus component level of analysis across subjects. Across sources, we found a high degree of heterogeneity across occipitoparietal alpha components in terms of their amplitude-distance correlation coefficients, ranging from −0.84 to + 0.93 with mean at 0.2 (see Fig. 6A). A permutation test showed that the observed standard deviation (0.527) of amplitude-distance correlation coefficients across components is highly unlikely to occur by chance (permuted mean standard deviation = 0.3773; *p* = 0.000056), see Fig. 6B), showing that there is a highly significant variability across occipitoparietal alpha components in terms of their SSVEP-PtP amplitudes as a function of flicker-IAF distance.

In order to ensure that this variability of evoked response patterns across sources was not confounded by an unequal distribution of sources across subjects, we focused on these subjects from whom two or more sources were analysed (10/13). 50% of these showed one variation of SSVEP-PtP relative to flicker-IAF distance out of the possible three (as illustrated in Fig. 1), 30% showed two variations, and 20% showed three variations. The range of amplitude-distance correlation coefficients that were categorised as exemplary of the superposition model (variation 2, Fig. 1) was set at r = −0.198–0.198, as this was the range of amplitude-distance correlation coefficients that were achieved by chance from a permutation test with 1000 repetitions on shuffled SSVEP-PtP values across the flicker conditions. Hence, the variability in the amplitude-distance correlation coefficients existed within-subjects.

Furthermore, we conducted a complementary analysis using a canonical FFT approach to further investigate our results using the SSVEP-PtP measure. We firstly compared our results with the results gained from the nearly identical analytical pipeline, with the only difference being the use of the evoked FFT approach to characterise the amplitude of our evoked responses to rhythmic flicker, rather than the SSVEP-PtP. Again, we found large variability in the amplitude-distance correlation coefficients across sources (standard deviation = 0.4365), with a trend to significance for this finding as shown via a permutation test (permuted mean standard deviation = 0.3760; *p* = 0.0548) (see Supplementary Fig. S2). In order to analyse potential confounds of this FFT-based complementary analysis, we investigated whether the assumptions of the FFT approach were fulfilled. Namely, a further analysis looking into the waveform shapes of our SSVEPs showed the existence of non-sinusoidal waveforms in our data, investigated by comparing the power at the first harmonic frequency with the fundamental frequency (i.e., flicker frequency) (see Supplementary Fig. S3). Some sources showed higher power at the harmonic than at the fundamental frequency; this difference in power was also variable across sources within subjects, with some sources showing a higher power at the harmonic and other sources showing a higher power at the fundamental within the same subject. Furthermore, the relative power at the fundamental versus harmonic was variable across flicker frequency conditions for any one source. This variability indicates not only the presence of both sinusoidal and non-sinusoidal waveform components within subjects, but also changes in waveform shapes depending on flicker frequency. These findings could explain the reduced significance of the result via the FFT approach: the assumption of sinusoidal waveforms in the FFT approach was not met. These results deemed the use of the peak-to-peak amplitude as the appropriate measure and further validated our findings.

This point was exemplified further in a supplementary analysis S4, looking into the statistical significance of the evoked responses (using an FFT approach) to flicker across the chosen components, expressed as z-scores (see Supplementary Figure S8). On average across components, a significant (*p* > 0.05) evoked response at the fundamental frequency was found for 4.31 out of the 8 tested flicker frequency conditions (with a standard deviation of 1.71). All components showed a significant evoked response to flicker in at least one flicker condition. Importantly, for some components in some conditions, the evoked amplitude of the second harmonic beyond noise was larger than that at the fundamental frequency, further illustrating the presence of non-sinusoidal waveforms in our data and hence the failure to meet the FFT-based assumption of sinusoidal waveforms. On average across components, a significant evoked response at the second harmonic was found for 2.91 out of the 8 tested flicker frequency conditions (with a standard deviation of 1.42).

A simulation analysis investigated the resistance of our analysis approach to outliers. With increasing outlier size added to simulated component data of SSVEP-PtP amplitudes across stimulation conditions, the correlation coefficient showed a change as the outliers increased in size over time, reaching a plateau at around 0.15–0.55 (depending on to how many components outliers were added), as the effect of the outliers reached a ceiling (see Supplementary Fig. S4). When directly comparing the results of our analysis pipeline on simulated data driven by our hypotheses (i.e., with ‘true’ linear trends in the data) with data driven by outliers, the resulting Z-scores for the ‘true’ data were consistently much larger than those obtained with the outlier data, which results in highly significant *p*-values for the hypothesized effect (average *p*-value = 0.00006, close to the actual result in the real experiment) and *p*-values which are often not significant for the outlier condition (average *p*-value = 0.02). This is despite the fact that both conditions were having the same average effect on the correlation coefficient of individual “components”. An example of one resulting distribution is shown in Supplementary Fig. S5. This result represents the ‘worst case’ scenario of outliers, as outliers were added to the last (most sensitive) point; fewer outliers or a different position would result in a weaker effect of the outliers.

**Figure 7:**
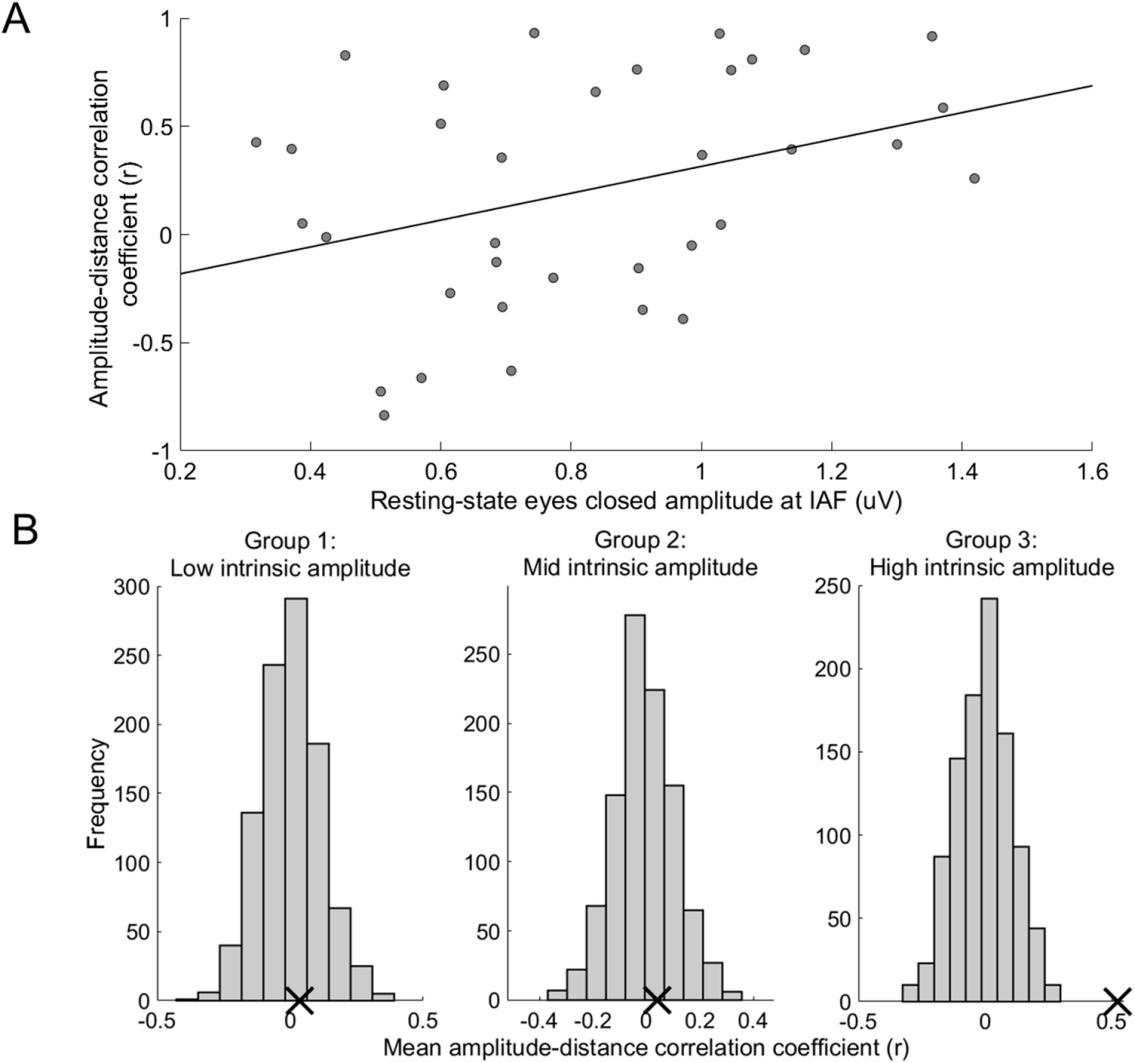
The relationship between the amplitude-distance correlation coefficients of occipitoparietal alpha oscillations and their amplitude at IAF at resting-state eyes closed. (Panel **A**). The linear mixed model analysis showed a significant predictive relationship of intrinsic IAF amplitude at resting-state eyes closed on the amplitude-distance correlation coefficient across all components (F(1,33) = 7.214, *p* = 0.011, slope estimate = 0.714, adjusted R^2^ = 0.485). A least squares best fit line is shown. (Panel **B**). Histograms of the permuted distribution of mean amplitude-distance correlation coefficients, grouped by intrinsic amplitude. This significant relationship is driven by the relatively higher intrinsically powered oscillators, as shown via three permutation tests. The 35 chosen components were split into three groups based on their amplitude at resting-state eyes closed at the IAF: low (amplitude < 0.62 uV), mid (amplitude between 0.62 and 0.972 uV) and high (amplitude > 0.972 uV). A permutation test was conducted on each group’s components, finding the distribution of the mean amplitude-distance correlation coefficient expected under the null hypothesis, permuted 1000 times. The permutation tests on Group 1 and Group 2 – i.e., components with low and mid resting-state eyes closed amplitude at IAF- showed that our observed mean amplitude-distance correlation coefficient (CC) for either of these groups lies well within the range expected by chance (Group 1: observed mean CC = 0.036, *p* = 0.379; Group 2: observed mean CC = 0.040, *p* = 0.340). Group 3 however, consisting of the components with high amplitude at IAF during resting-state eyes closed, had an observed mean CC that lies well outside the permuted distribution expected by chance (observed mean CC = 0.523, *p* < 0.001, surviving the Bonferroni correction for multiple comparisons). Observed mean CC are shown for each group with a black cross and permuted distributions of mean CC are shown for each group with grey bars.

To explore whether the source variability of SSVEPs can be explained by each source’s maximal amplitude at IAF during rest, we also studied the relationship between the amplitude-distance correlation coefficients and the intrinsic resting-state eyes closed amplitudes at the peak IAF across components. The intrinsic alpha frequency of each source is shown in Supplementary Fig. S6; a range of 8.7–10.9 Hz can be seen across our chosen components. A linear mixed model analysis showed a significant predictive relationship of intrinsic amplitude at resting-state eyes closed on the amplitude-distance correlation coefficient across all components (F(1,33) = 7.214; *p* = 0.011, R^2^ = 0.500; slope estimate = 0.714; standard error = 0.266; 95% CI [0.173 1.254]; see Fig. 7A). Visual inspection of the data indicated that the significant predictive relationship was driven by high alpha amplitude components. Therefore, to further investigate this trend, permutation tests on each of the three groups of components based on their resting-state eyes closed amplitude at IAF were performed. This showed a significant result only for those components with the relatively highest amplitude at IAF during resting-state eyes closed (i.e., an amplitude > 0.972uV): our observed mean amplitude-distance correlation coefficient of 0.523 for these high intrinsically powered components lay well out of the distribution of mean amplitude-distance correlation coefficients expected by chance, *p* < 0.001, surviving Bonferroni correction (see Fig. 7B, ‘Group 3’). The low (see Fig. 7B ‘Group 1’) and mid (see Fig. 7B, ‘Group 2’) intrinsically powered components showed mean amplitude-distance correlation coefficients that lay within the range of values expected by chance and were not statistically significant (Group 1: observed mean amplitude-distance CC = 0.036, *p* = 0.379; Group 2: observed mean amplitude-distance CC = 0.040, *p* = 0.340).

## Discussion

The question of the current study was whether distinct occipitoparietal alpha sources show heterogeneity in their evoked responses to rhythmic flicker relative to IAF. We found, to the best of our knowledge for the first time, remarkable variation across sources. This result provides first insight into the possibility of variability across alpha oscillations in terms of how/whether they interact with or contribute towards evoked responses.

Across alpha oscillatory sources, we report extreme and statistically significant variability in the amplitude-distance correlation coefficient measure (ranging from −0.84 to + 0.93), which illustrates the high variability that exists at the tICA-derived source-level of occipitoparietal alpha oscillators in terms of their evoked response patterns to visual flicker relative to IAF. As sources were identified using tICA (and hence sources are simply reflected via an electrode weighting), it isn’t possible to draw any conclusions as to whether the SSVEPs are generated directly from the identified alpha sources themselves. Our approach of estimating components based on concatenated resting state data with flicker data further exemplifies this point: the possible mixing of sources into individual components means that some of our chosen components could have been a mix between endogenous alpha sources at rest with simple summated ERPs during flicker. What we can conclude is that our reported variability likely reflects different interactions/contributions of alpha generators on/towards the evoked responses.

As our Pearson’s correlation-based outcome measure of amplitude-distance correlation coefficients, based on between 5 and 8 data points (depending on where the IAF lay on the scale of flicker frequencies tested), was prone to outliers, we controlled for this potential confound by permutation analysis, which is equally sensitive to outliers; our reported standard deviation (0.527) of correlation coefficients across sources lies significantly out of the range of chance (permuted mean standard deviation = 0.3773; *p* = 0.000056) and demonstrates the reliability of our finding. Our control analysis on simulated data verified this, showing that our analysis pipeline is much more sensitive to subtle positive or negative trends in the data, than it is to outliers. A further control analysis (Supplementary Analysis S3), looking at the distribution of noise in SSVEPs across components showed that the signal-to-noise ratio (SNR) across components is not significantly different. Hence, the reported variability in the amplitude-distance correlation coefficients is unlikely to be driven by different distributions of noise across components; rather, the variability represents differences in amplitude of meaningful SSVEPs elicited by flicker at each frequency relative to IAF. Furthermore, amplitude-distance correlation coefficients are differently distributed across subjects, i.e., all subjects with several IAF sources of interest have sources of different SSVEP responses relative to IAF, namely correlation coefficients. This result demonstrates that variability of SSVEP responses across sources is not confounded by variability across subjects. Finally, our correlation coefficient outcome measure based on the SSVEP peak-to-peak amplitude, was not confounded by ‘problematic’ (i.e., often unfulfilled) assumptions on the oscillatory behaviour of sources, which are typical for other analytical approaches such as the canonical FFT approach; additional analyses demonstrated non-sinusoidal waveforms in our data validates our use of the SSVEP peak-to-peak amplitude approach (see Supplementary Analyses S1 and S4). A control analysis (Supplementary Analysis S4) investigated the statistical significance of our evoked responses to flicker across components, by expressing the evoked amplitude as z-scores. All components showed a statistically significant evoked response to flicker in at least one condition. Some components, in some conditions, showed a higher amplitude z-score at the second harmonic than the fundamental flicker frequency. This illustrates the presence of non-sinusoidal oscillations in the data, which has been previously shown and discussed in terms of the repercussions on the validity of results as acquired via FFT-based analyses^66^. Even with some components in some conditions showing weak evoked responses beyond noise, we showed via our simulation analysis that the group level permutation test is sensitive enough to show an effect that would not be present if all the evoked responses were just noise. These points together illustrate not only the validity of the approach taken (namely peak-to-peak amplitude of evoked responses as opposed to evoked FFTs) but also the reported variability in the evoked responses to flicker relative to IAF across alpha oscillatory sources.

The current debate as to which mechanisms underlie SSVEPs is strictly dichotomous: one body of research argues for a neural entrainment mechanism^8,12,14^, whereas another argues for superposition^11,26^. Our findings of variability in the evoked response patterns relative to IAF across distinct occipitoparietal alpha sources show that different sources interact differently with evoked responses to visual flicker relative to the endogenous alpha frequency and provide first indication that SSVEPs can reflect a myriad of different interactions with underlying alpha generators.

Three patterns of response to rhythmic flicker relative to IAF were identified. Firstly, we found that approximately one-third of our occipitoparietal alpha sources showed a maximal evoked response to rhythmic flicker at IAF, decreasing in amplitude with increasing flicker frequency distance from IAF (Fig. 1, ‘Variation 1’). This evoked response pattern, illustrating a non-linear evoked response around the IAF, would be expected under an SSVEP mechanistic model whereby the flicker interacts with the endogenous rhythms.

One such model is the neural entrainment model, positing that the phase of the endogenous oscillator is gradually shifted into alignment with the rhythmic flicker presented at the IAF. This phase-alignment gets weaker with increasing flicker frequency distance from IAF, leading to a maximal evoked response to IAF stimulation that decreases as a function of rhythmic flicker frequency distance from IAF. On an anatomical level, alpha oscillations are subjected to a thalamic pacemaker^46–48^, showing coherence with activity in the thalamic lateral geniculate nucleus in animals^46,49^. Coherence between alpha amplitude and thalamic metabolic activity in human studies provides further support for the thalamic involvement in alpha oscillations^50–53^. Activation of glutamate receptors in cat LGN has been found to generate alpha oscillations via high-threshold bursting^54,55^. Visual flicker may entrain certain sources of alpha oscillatory activity by driving burst activity in the LGN, leading to synchronised thalamocortical activity at the frequency of flicker. This ‚driving ‘ would be strongest at flicker frequencies closest to the system resonance frequency, i.e., the IAF.

As we focused on evoked activity only (to avoid the problem of spectral overlap when disentangling evoked from endogenous alpha activity), we are unable to draw any firm conclusions as to whether this pattern of evoked response represents entrainment or other potential mechanisms.

The second pattern of response that we found was one of no change in evoked response to rhythmic flicker relative to IAF (or a response pattern reflecting 1/f decay) (Fig. 1, ‘Variation 2’). This finding could be expected when assuming a superposition model of SSVEPs, i.e., no interaction between the ongoing oscillator and the external rhythmic flicker and hence no change in the evoked response to rhythmic flicker relative to IAF. Our control analysis looking into the distribution of noise in SSVEPs across components (Supplementary Analysis S3) showed a similar signal-to-noise ratio across all components, regardless of their amplitude-distance correlation coefficient (see Fig. S7). Hence, a near-zero amplitude-distance correlation is indicative of a meaningfully elicited SSVEP that remains independent from the intrinsic oscillatory system frequency, i.e., superposition. From an anatomical viewpoint, the model of superposition would assume no interaction or ‘driving’ of thalamic burst activity to the cortex via the flicker; rather, the intrinsic thalamocortical oscillatory system would remain independent, and the SSVEPs would simply represent evoked cortical neuronal responses to visual stimulation. Noteworthy here is the possibility of some of our chosen components representing the mu rhythm as opposed to intrinsic alpha oscillators due to volume conduction and the limitations of source localisation in terms of source specificity. In this case, a change in SSVEP amplitude to flicker within the alpha range wouldn’t be expected. Another possibility could be that the endogenous alpha oscillators simply ceased to exist at the time of flicker, which would also lead to a non-changing evoked response across flicker frequencies.

Our third pattern of evoked response was a minimal evoked response to IAF rhythmic flicker that increased with flicker frequency distance from IAF (Fig. 1, ‘Variation 3’). Approximately one-third of components showed this pattern of response and neither the classic entrainment model, nor the superposition model, would be able to account for this pattern. We propose two alternative models which can account for this evoked response pattern: a model of “inhibitory-phase entrainment”, and a model of “destructive interference”. Our first model posits entrainment of an endogenous alpha oscillator, such that the phase and frequency match the flicker, but with the critical phase of the visual flicker (e.g., light-on) aligning with the inhibitory phase of the alpha wave. This would lead to an overall minimal evoked response, which would increase in amplitude to rhythmic flicker frequencies further away from the IAF, as the inhibitory-phase entrainment would become weaker, and flickers would be more likely to slip into non-inhibitory phases. Anatomically, this model could be highly similar to that of traditional entrainment of thalamic activity to flicker, but with a specific entrainment of the thalamic activity to the inhibitory phase of the flicker. One postulated functional role of such inhibitory-phase entrainment of thalamic burst activity could be the suppression of cortical processing of distractor visual input. This model could be potentially testable in future work through the variation of the flicker duty cycle, for example. Our second model of destructive interference posits that the minimal evoked response to rhythmic flicker at IAF could occur due to *chance* inhibitory-phase alignment of the rhythmic flicker to the inhibitory phases of the ongoing oscillator. Crucially, this chance alignment would occur consistently across time due to the close periods of the rhythmic flicker at IAF and the ongoing oscillator. Under this model the visual flicker will hit the inhibitory phase of the alpha oscillator by chance an equal number of times regardless of the frequency, but when the frequencies closely match, the visual flicker will hit the inhibitory phase many times in a row, rather than sporadically. This chain of subsequent matching to the inhibitory phase might lead to a net reduction of SSVEP amplitude, which is not caused by occasionally hitting the inhibitory phase in isolation. This model would account for an overall minimal evoked response at IAF rhythmic flicker that increases with flicker frequency distance from IAF.

With the use of a temporal ICA, we were able to separate the distinct sources and identify their responses to rhythmic flicker relative to IAF individually, as opposed to taking the traditional scalp-level approach where the activity of all individual alpha oscillators is averaged together. This more refined view of the amplitude changes of distinct alpha oscillators allowed us to see variability in the evoked response relative to IAF across distinct sources that is lost at the scalp-level. A recent study^7^, investigating the tracking of quasi-rhythmic versus rhythmic alpha stimulation in terms of phase synchronisation across various cortical regions, reported a small increase in coherence for rhythmic versus quasi-rhythmic alpha stimulation in primary visual cortices, other cortical regions however did not show any change in coherence. This finding potentially corroborates ours of spatial variability in response to flicker near the oscillatory resonance frequency; some oscillatory sources may be entrained to alpha flicker stimulation, others may remain independent. Furthermore, it is possible that any larger differences in coherence or behavioural effects for rhythmic versus arrhythmic alpha stimulation may have been masked by the confound of flicker frequency distance from IAF.

As our analyses focus solely on evoked responses from tICA-derived sources, the possibility of a largely incommensurate set of SSVEP generators existing in parallel to our sources of alpha oscillatory activity stands. Hence, we are unable to draw any firm conclusions as to whether SSVEPs are themselves generated by multiple mechanisms, but rather that our reported variability likely reflects different types of interaction between SSVEPs and alpha generators across different spatial sources.

Our exploratory analysis, investigating the relationship between the eyes-closed rest amplitude at IAF of each alpha oscillator and the amplitude-distance correlation coefficient, showed that the highest intrinsically powered sources showed exclusively positive correlation coefficients, i.e., a minimal evoked response to rhythmic flicker at IAF that increased with flicker frequencies further away, a finding shown to be highly statistically significant via grouped permutation testing. Mid- and low-intrinsic amplitude sources did not show any consistent pattern of evoked response to rhythmic flicker relative to IAF. This suggests that the variation across distinct occipitoparietal alpha sources in terms of their evoked response to rhythmic flicker relative to IAF could be partially explained by their intrinsic amplitude. A provisional explanation for this is that the strongest endogenous oscillations, although weaker during visual stimulation, remain present and are unaffected by the visual flicker, i.e., are not entrained, but rather persist and cause a frequency-specific reduction in amplitude of the SSVEPs. A previous study using simultaneous invasive recording and 10 Hz-pulsed cortical electrical stimulation showed that the effect of stimulation depends on the power of the pre-existing oscillations: during strong neural oscillations (eyes closed) the stimulation had little effect, but for weaker oscillations the stimulation evoked a response matched to the stimulation frequency^56^. Although that study used electrical stimulation rather than visual flicker, the relationship between endogenous power and entrainment may be similar. This may explain why none of the strongest alpha sources here showed an evoked response pattern that would be expected under the entrainment model. In fact, our finding of the endogenously strongest alpha components showing exclusively positive correlation coefficients may provide further support for our model of destructive interference, from a signal-to-noise ratio standpoint. Relative to endogenously weaker components, the strongest components would have a more defined phase, which would enable maximal interference with SSVEPs (and hence a maximally reduced SSVEP amplitude) during IAF stimulation.

### Methodological issues, limitations, and strengths

#### Sample size

A notable limitation of our study is our sample size (*N* = 16 acquired; *N* = 13 following subject removal). However, we were able to account for this limitation, at least to some extent, via both our power-increasing component-based analysis approach including 35 alpha oscillatory components distributed across these 13 subjects and our use of permutation tests to determine the statistical significance of our results. Notwithstanding, our results need replication in a larger sample.

#### Experiment, assessment

Another noteworthy feature of our study is that subjects performed the experiment in a supine position. This was not only to allow for a second fMRI dataset acquired from the same subjects, but also provided compatibility with the wealth of previous resting state fMRI studies, where participants are supine. Body position can significantly alter the resting cortical state^57,58^: amplitude changes in the alpha band during resting-state eyes open are sensitive to body position, with the supine position inducing cortical inhibition^59^. The supine position of our subjects may be an important factor to take into consideration when comparing our results to those of other studies with subjects in a seated position. However, the studies mentioned above focused only on the resting state; future work is needed to look into the effect of seated versus supine position on amplitude changes during rhythmic flicker stimulation. A further drawback in our study design was our lack of control over subject fatigue. Previous studies have shown a strong negative relationship between subject sleepiness and resting state alpha power^60^ as well as significant changes in SSVEP amplitudes under conditions of fatigue^61^. Future studies may look at the effect that fatigue has on SSVEP amplitude across alpha flicker conditions at different sources of intrinsic alpha activity.

#### EEG source localisation: inherent weaknesses

The solution of the inverse problem in EEG, i.e., the calculation of the position of multiple simultaneously active equivalent dipoles based on electrode potentials, is inherently ill-posed; there is no one unique solution^62^ and this limitation must be kept in mind when considering our results. However, a major advantage of our approach of first temporally unmixing the data into components leads to data that is significantly more dipolar than the raw mixed data, which significantly increases the accuracy of source localisation^63,64^. Furthermore, our analyses allowed for some flexibility in terms of spatial source localisation inaccuracy, having only the very broad criteria of being located in occipital or parietal cortices.

## Conclusion

In conclusion, our findings reveal heterogeneity in the evoked response patterns of distinct occipitoparietal alpha oscillators to rhythmic flicker relative to IAF. This variability illustrates that different sources of alpha oscillatory activity deal differently with flicker-evoked responses relative to endogenous frequency. Observing the responses of independent alpha sources to rhythmic flicker may be a more promising approach to understanding the mechanisms that lead to the generation of SSVEPs.

## Supplementary Information

Supplementary Information 1.

Supplementary Information 2.

Supplementary Information 3.

Supplementary Information 4.

Supplementary Information 5.

Supplementary Information 6.

Supplementary Information 7.

Supplementary Information 8.

Supplementary Information 9.

## Data Availability

Data is available via request to the first/corresponding author (rachel.nuttall@tum.de). Third party software and code were utilised for the completion of this study. All relevant software details (versions, links to code repositories, etc.) are appropriately cited in text and in the list of references.

## Abbreviations

SSVEP: Steady-state visually evoked potential
SSVEP-PtP: The peak-to-peak amplitude of the steady-state visually evoked potential
IAF: Individual alpha frequency
Flicker-IAF distance: Absolute distance of the flicker frequency from the IAF
Amplitude-distance CC: The correlation coefficient between the peak-to-peak amplitude of the average steady-state visually evoked potential and the absolute distance of the flicker frequency from the IAF
