# Supplementary material for: Evoked responses to rhythmic visual stimulation vary across sources of intrinsic alpha activity in humans
Source: Sci Rep. 2022 Apr 8;12:5986. doi: 10.1038/s41598-022-09922-2 (PMC8993822; doi:10.1038/s41598-022-09922-2)
Supplement: Supplementary file 1 — Supplementary Information 1. [file 41598_2022_9922_MOESM1_ESM.docx]

**Supplementary Material for:**

**Title**

**Evoked responses to rhythmic visual stimulation vary across sources of intrinsic alpha activity in humans**

**Author names and affiliations**

Nuttall, R*^1,2,6^; Jäger, C^1,2^; Zimmermann, J^1,2^; Archila-Melendez, M. E^1,2^; Preibisch, C^1,2^; Taylor, P^3,4,7^; Sauseng, P^5^; Wohlschläger, A^1,2^; Sorg, C^1,2**^; Dowsett, J^1,2,4,7**^

^1^Technical University of Munich, School of Medicine, TUM-Neuroimaging Center, Munich, Germany

^2^Technical University of Munich, School of Medicine, Department of Neuroradiology, Munich, Germany

^3^Ludwig Maximilian University, School of Medicine, Department of Neurology, Munich, Germany

^4^Ludwig Maximilian University, School of Medicine, German Center for Vertigo and Balance Disorders, Munich, Germany

^5^Ludwig Maximilian University, Munich Center for Neurosciences – Brain & Mind, Munich, Germany

^6^Technical University of Munich, Department of Anesthesiology and Intensive Care, Munich, Germany

^7^Ludwig Maximilian University, Department of Psychology, Munich, Germany

** Both authors contributed equally

**Content list:**

Supplementary Analysis S1

Supplementary Analysis S2

Supplementary Analysis S3

Supplementary Analysis S4

Supplementary Figures S1-8

**Supplementary Analysis S1:**

Further analyses were performed to infer the validity of our results based on our decision to use SSVEP-PtP to characterise the amplitude of evoked responses as opposed to the traditional FFT approach. Firstly, we repeated our analysis pipeline with the only change being the use of an evoked FFT analysis as opposed to the SSVEP-PtP. The rhythmic flicker stimulation data of each of our 35 chosen components across all three blocks were segmented into non-overlapping 4 second segments and the mean 4 second data segment entered into an FFT, giving an FFT spectra per rhythmic flicker condition per component. The absolute amplitude at the flicker frequency per rhythmic flicker condition was extracted and converted into signal-to-noise ratio, as previously defined^65^. The ratio between the absolute amplitude at the flicker frequency to the average absolute amplitude at the neighbouring frequencies (+/-2Hz, excluding +/-.5Hz directly around the flicker frequency) was computed. These evoked FFT amplitude SNR values across flicker frequency conditions were correlated via a Pearson’s correlation with the flicker frequency as an absolute distance from the IAF, giving us our outcome measure of an amplitude SNR-distance correlation coefficient per component. The standard deviation of the amplitude-distance correlation coefficients across components was analysed for statistical significance via a permutation test with 1000 repetitions (the procedure for performing the permutation test was the same as in the main SSVEP peak-to-peak amplitude analysis). As can be seen in Supplementary Fig. S2, in contrast to our peak-to-peak amplitude approach in our main analysis, the standard deviation of the evoked FFT amplitude SNR-distance correlation coefficients (0.4365) was marginally significantly outside of the range of standard deviation values expected by chance (permuted mean standard deviation = 0.3760; p = .0548). This difference in results obtained from the two analysis approaches suggests that the sinusoidal waveform assumption of the evoked FFT analysis may not be met.

To further investigate this, we looked at the waveform shapes of our evoked responses by taking, for each flicker frequency condition for each component, the absolute amplitude in the evoked FFT at the first harmonic frequency of the flicker subtracted from the fundamental flicker frequency absolute amplitude. For sinusoidal evoked responses across conditions and components, this approach should give us only positive values. For non-sinusoidal evoked responses or a variety of sinusoidal and non-sinusoidal responses, we should see negative values remaining. As can be seen in Supplementary Fig. S3, our finding of both positive and negative values remaining suggest the existence of non-sinusoidal waveforms in our data, corroborating our choice of using the SSVEP peak-to-peak amplitude approach as opposed to the evoked FFT approach.

**Supplementary Analysis S2:**

To quantify how resistant to outliers our main analysis pipeline is, simulated data was created to model various effects. Firstly, a null data set was created; this consisted of 35 components with 8 data points each, corresponding to the 8 SSVEP amplitudes at each frequency. Each data point was assigned a random value between 0 and 1; this represents the random variability we might see due to noise. From this null data set two conditions were created, firstly a “true effect” which models our hypothesised effect. Each component was randomly assigned to one of three conditions: either a positive bias, a negative bias or was left as is (no effect). Positive bias consisted of a small linear trend added to the random values and negative bias consisted of a small negative linear trend. The analysis was then run on these values: the correlation for each component was calculated, the standard deviation of these correlation coefficients was taken, and then the 8 “amplitudes” for each component were randomly shuffled and the procedure was repeated 1000 times to create the null distribution. This entire procedure was run with various sizes of bias until the resulting effect was similar to the effect seen in the real data, i.e., the resulting p-value was approximately 0.00005. Specifically, this was 8 values in a linear trend from zero to 0.53 (or zero to -0.53 in the case of the negative bias) which was added to the 8 random values. This served as a model of the hypothesized effect: either a positive or negative trend in SSVEP amplitudes relative to individual alpha frequency.

For the simulated outlier condition random null data was created, following the same procedure as outlined previously. Next, the last data point (which is maximally sensitive to outliers) was replaced with either a positive outlier, or a negative outlier. Outliers were added to varying numbers of components, i.e., firstly to every component, then to every other component, then every third. This data was then run through the same analysis pipeline many times with various sizes of outlier. For each, the average absolute change in correlation for each component was calculated. Specifically, for each simulated “component”, the correlation coefficient of the initial random “null” data was subtracted from the correlation coefficient of the data with the outlier added. Supplementary Fig. S4 shows how this change in correlation evolves over time as the outliers get larger. The average absolute change in correlation eventually plateaus as the effect of the outliers reaches a ceiling. The only condition in which the average difference in correlation is able to reach the same size as the simulated “true effect” is when an outlier is added to every component. If an outlier is added to every other component, or fewer, the average difference in correlation will never reach the equivalent level, regardless of how large the outliers are. Therefore, in the following steps outliers were added to every component.

Finally, we selected a value of outlier which resulted in the same average absolute change in correlation as the “true effect” data set. This value was selected to compare to the “true effect” data. The full analysis pipeline was then run on both types of data 100 times and the results were compared.

The results show that the “true effect” condition consistently gives much higher Z-scores than the outlier condition, which results in highly significant p-values for the hypothesized effect (average p-value = 0.00006, close to the actual result in the real experiment) and p-values which are often not significant for the outlier condition (average p-value = 0.02). This is despite the fact that both conditions were having the same average effect on the correlation coefficient of individual “components”. An example of one resulting distribution is shown in Supplementary Fig. S5. It is worth noting that this outlier condition had outliers at the last (most sensitive) point; outliers at a different position would result in a weaker effect of the outliers. This simulation is the “worst case” of outliers which can still result in the same average change in correlation as the simulated effect; fewer outliers would not reach the same average change in correlation coefficient and would not be comparable.

The conclusion we can draw from this simulated data is that this analysis pipeline is much more sensitive to subtle positive or negative trends in the data, than it is to outliers. With an equivalent average effect on the correlation coefficient of individual “components” (consisting of 8 data points each), the outliers are less likely to result in a significant effect on the standard deviation of correlation values. Outliers would need to be much larger to result in an equivalent low p-value and be present in every component. This does not rule out the possibility that the effect in the main experiment was driven by outliers, but it does suggest that this is far more likely to be due to our hypothesis. Specifically, that there is either a positive or negative trend in SSVEP amplitudes relative to individual alpha frequency.

**Supplementary Analysis S3:**

To control for the possibility of some components’ SSVEPs representing noise rather than a true elicited response to flicker, we calculated a conservative measure of signal-to-noise ratio of each SSVEP for each condition and component. For each flicker period in each condition, we randomly shuffled the component data and took a mean across shuffled flickers, to estimate a ‚noise‘ SSVEP for each condition. To get the signal-to-noise ratio of our SSVEPs, the peak-to-peak amplitude of the real SSVEP was divided by the peak-to-peak amplitude of the noise SSVEP. Supplementary Fig. S7 shows the signal-to-noise ratio of each component across frequency conditions. As can be seen in the figure, the SNR is not significantly different between the components.

**Supplementary Analysis S4:**

We investigated the statistical significance of the evoked responses to flicker across our chosen components. FFT spectra per rhythmic flicker condition per component were calculated as in Supplementary Analysis S1. The evoked amplitude for each component at each flicker frequency (both at the fundamental frequency and the second harmonic) was expressed as z-scores, calculated by taking the amplitude at the fundamental/second harmonic frequency and subtracting the mean of the surrounding frequencies (+/-2Hz, excluding +/-0.5Hz). This value was then divided by the standard deviation of those surrounding values. In Supplementary Figure S8, the z-scores of the evoked amplitude at the fundamental flicker frequency and the second harmonic can be seen. All components showed a significant evoked response to flicker in at least one condition. Importantly, the increased values at the second harmonic as compared with the fundamental frequency shown for some components at some flicker conditions further illustrates the presence of non-sinusoidal waveforms in our data and hence the inherent weakness in the use of a sinusoidal waveform-assuming FFT analysis approach as to the identification of the statistical significance of evoked responses to flicker that show non-sinusoidal waveform components.

**Supplementary Figures S1-7:
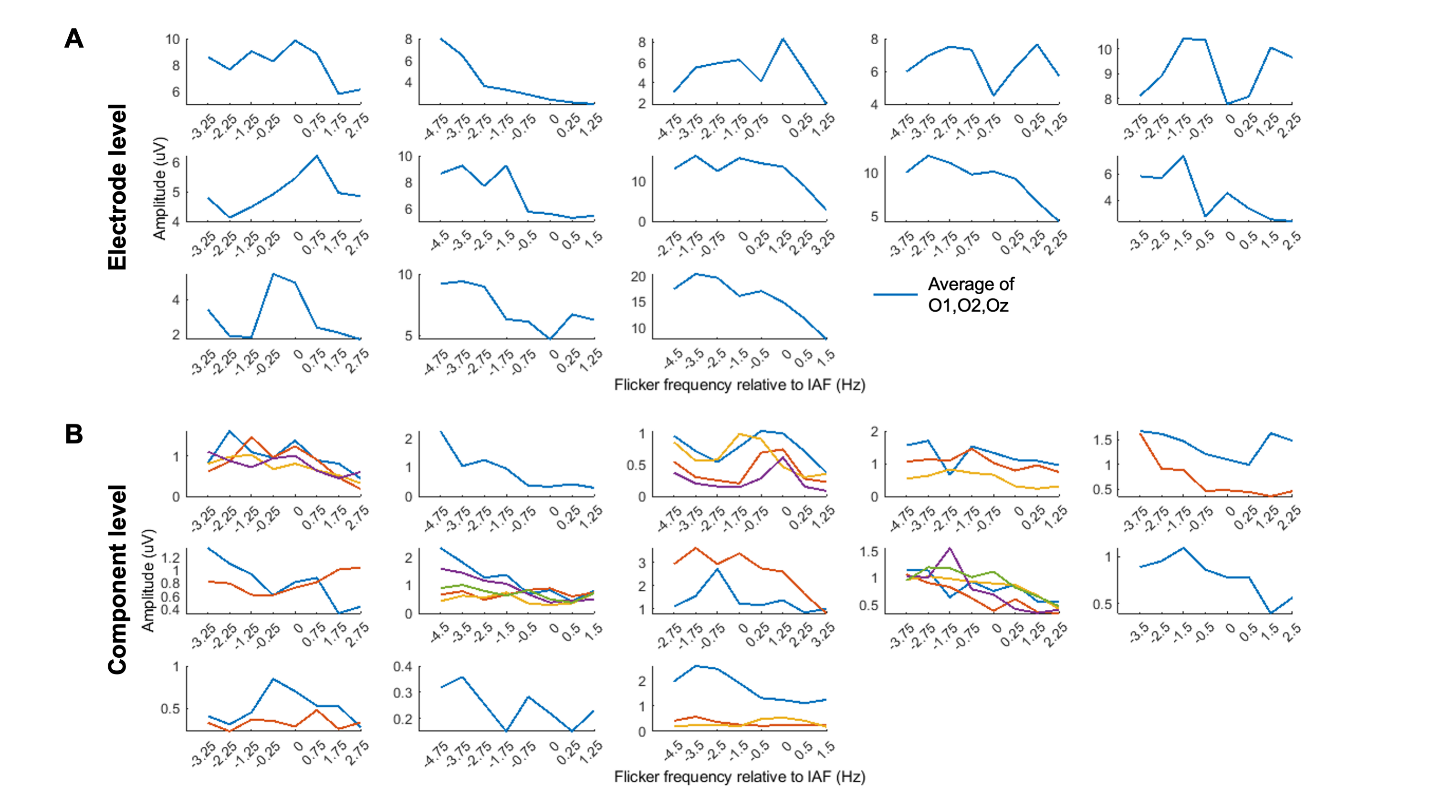
**

**Figure S1. Electrode versus component level evoked amplitude relative to IAF across subjects.** Panel A. Electrode level. For each of the 13 subjects, the average SSVEP peak-to-peak amplitude across electrodes O1, O2 and Oz is shown as a function of flicker frequency distance from IAF. Panel B. Component level. For each of the 13 subjects, the SSVEP peak-to-peak amplitude for each of the chosen components for that subject is shown as a function of flicker frequency distance from IAF.

**
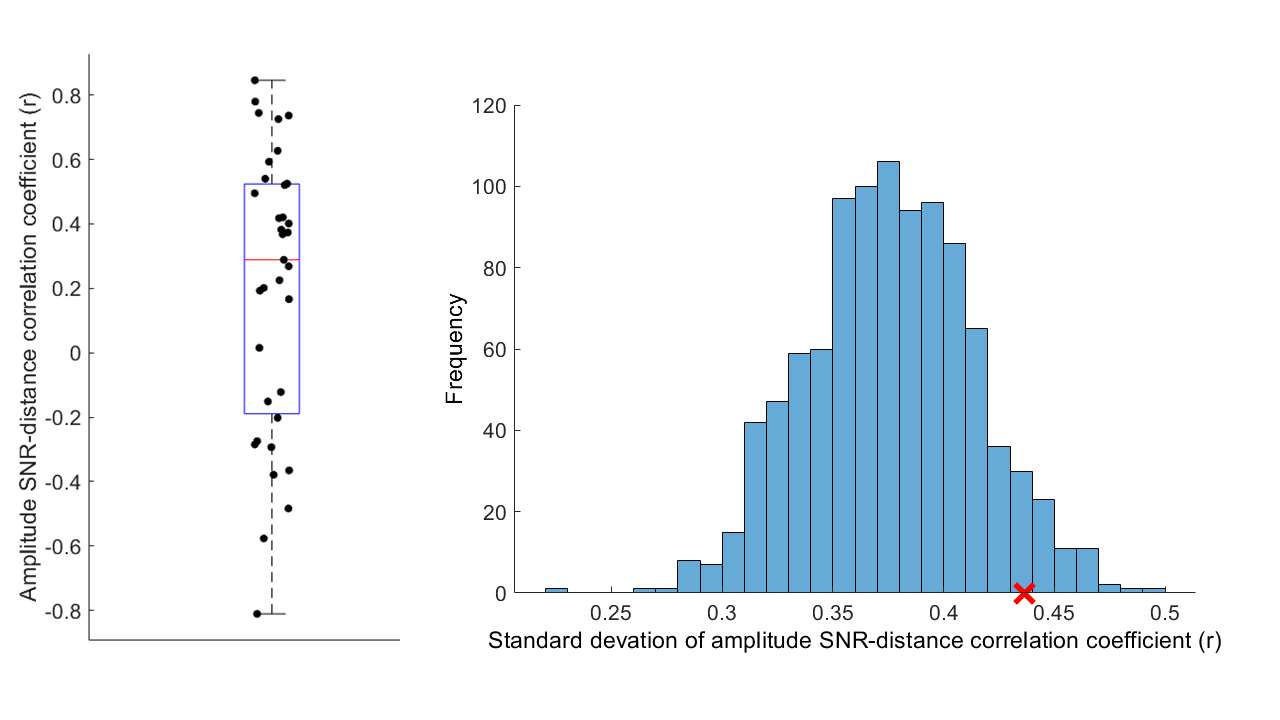
Figure S2. Variability in the visually evoked response of distinct occipitoparietal alpha oscillations to rhythmic flicker relative to IAF: Evoked FFT analysis approach.** Boxplot (left) illustrating the large variability in the amplitude SNR-distance correlation coefficients across the 35 alpha oscillatory components, with the horizontal red line representing the median, and the top and bottom edges of the blue box representing the 75^th^ and 25^th^ percentiles, respectively. Histogram (right) illustrating the results of the permutation test on the standard deviation of the amplitude SNR-distance correlation coefficients across all 35 alpha oscillatory components, which showed that our observed standard deviation value of 0.4365 (marked by a red cross) lies well outside of the range of standard deviation values likely to occur by chance (permuted mean standard deviation = 0.3760, shown in blue bars) retrieved from 1000 permutations, p = .0548.


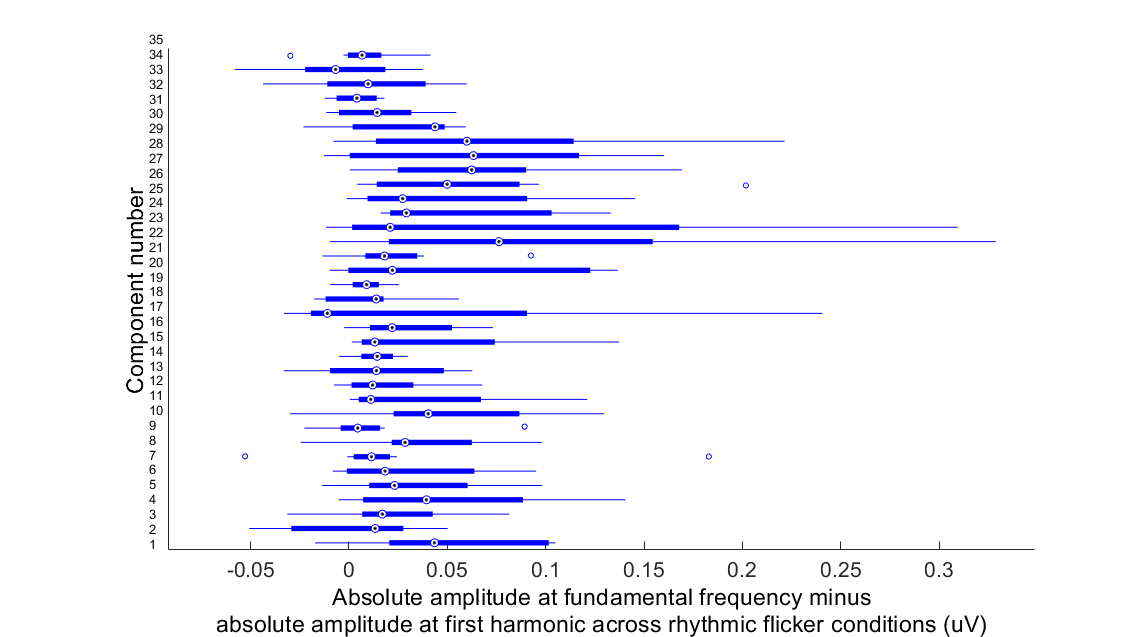


**Figure S3. Evoked waveform shape analysis.** Boxplots across all 35 alpha oscillatory sources demonstrating the difference in power at the fundamental frequency (i.e., flicker frequency) and the first harmonic frequency across the eight different flicker frequency conditions, calculated from the evoked FFT power spectra. Circles with blue dots inside indicate the median, and the left and right edges of the blue boxes represent the 25^th^ and 75^th^ percentiles. Thin blue lines on either side indicate the minimum and maximum values.


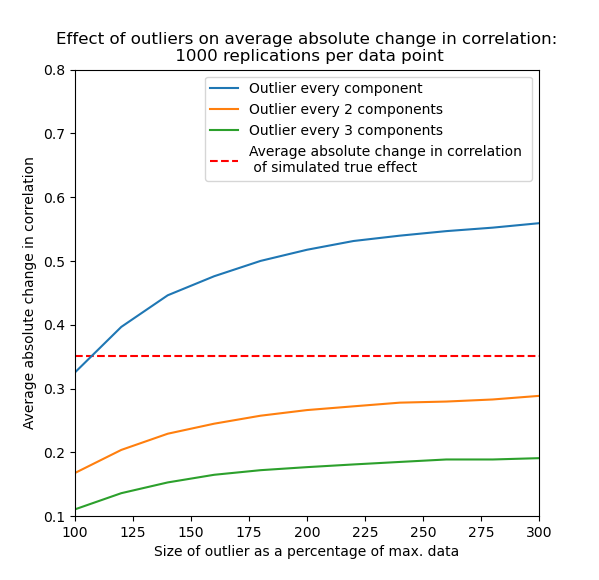


**Figure S4. The additional change in average correlation due to increasingly large outliers added to simulated data.** Outliers were added to every component, every other component, and finally every third component.


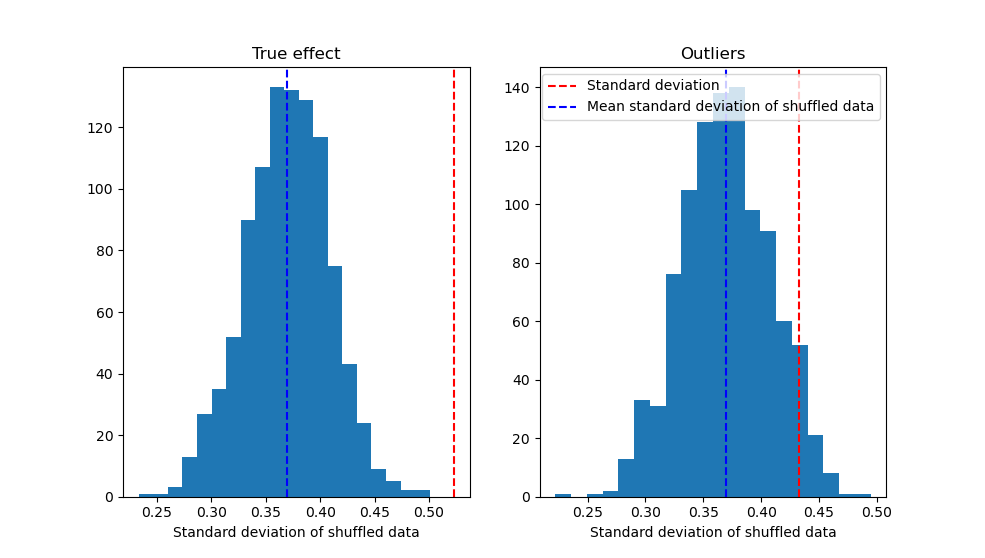


**Figure S5: Results of the experimental pipeline repeated for simulated trend (“true effect”) and outliers.** The size of the outliers were selected to result in the same average change in correlation. In this case p=0.00035 for true effect and p=0.3 for the outliers data.

**
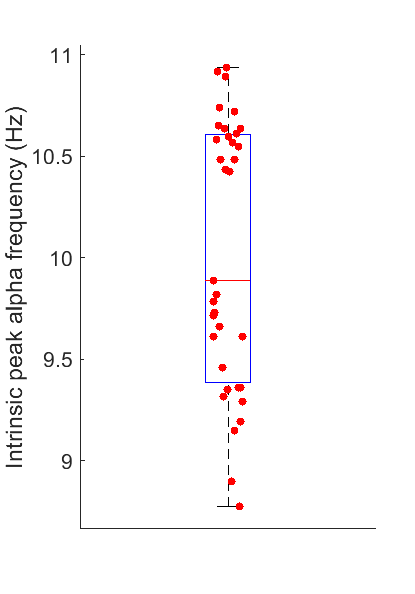
**

**Figure S6. Distribution of intrinsic peak alpha frequencies across chosen components.**

**
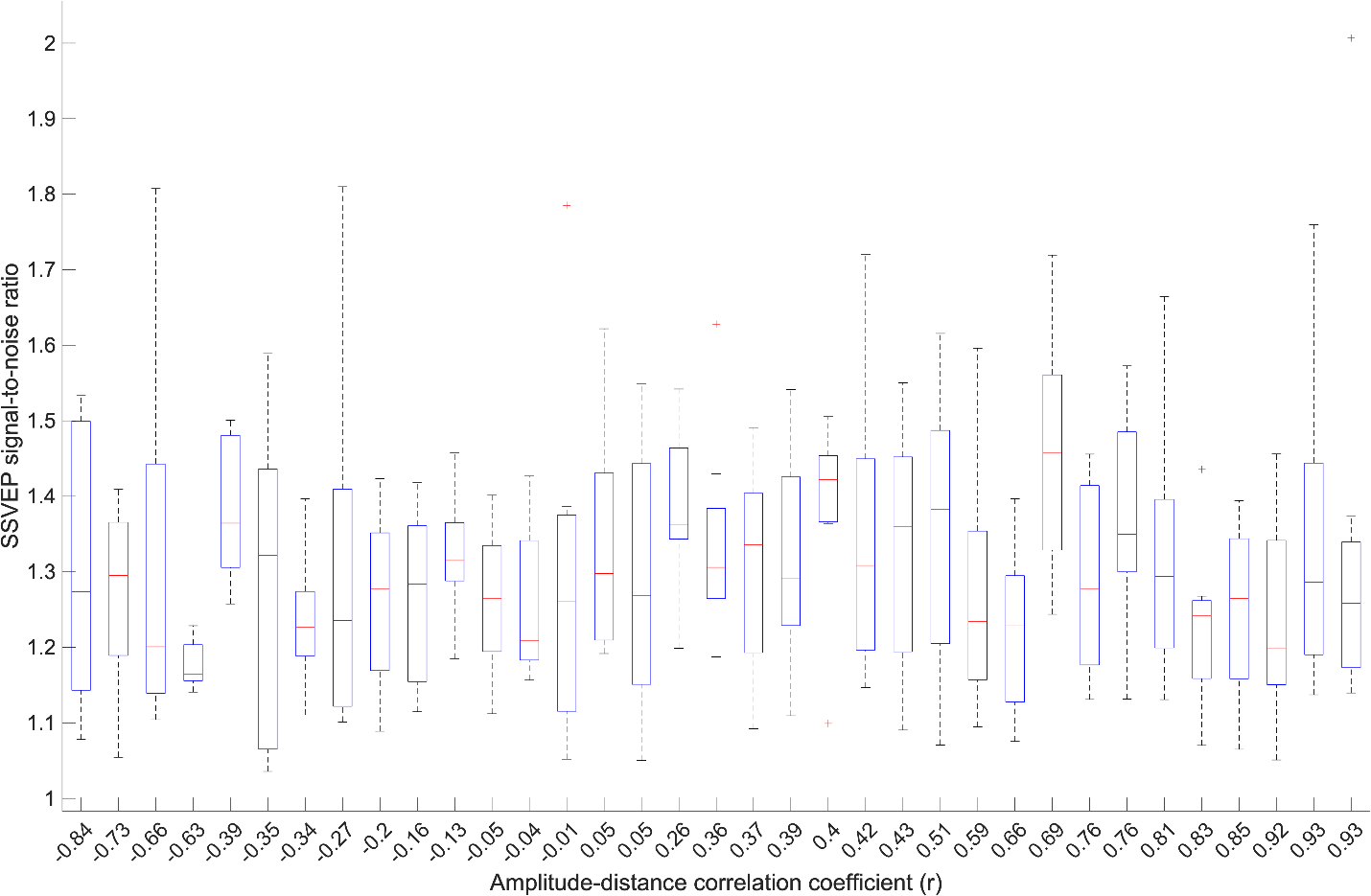
**

**Figure S7. Signal-to-noise ratio of SSVEPs across conditions for each component.** The signal-to-noise ratio (SNR) of the SSVEP across conditions is displayed for each component, labelled with each components’ amplitude-distance correlation coefficient. The SSVEP SNR is roughly similar across all components, regardless of their amplitude-distance correlation coefficient.


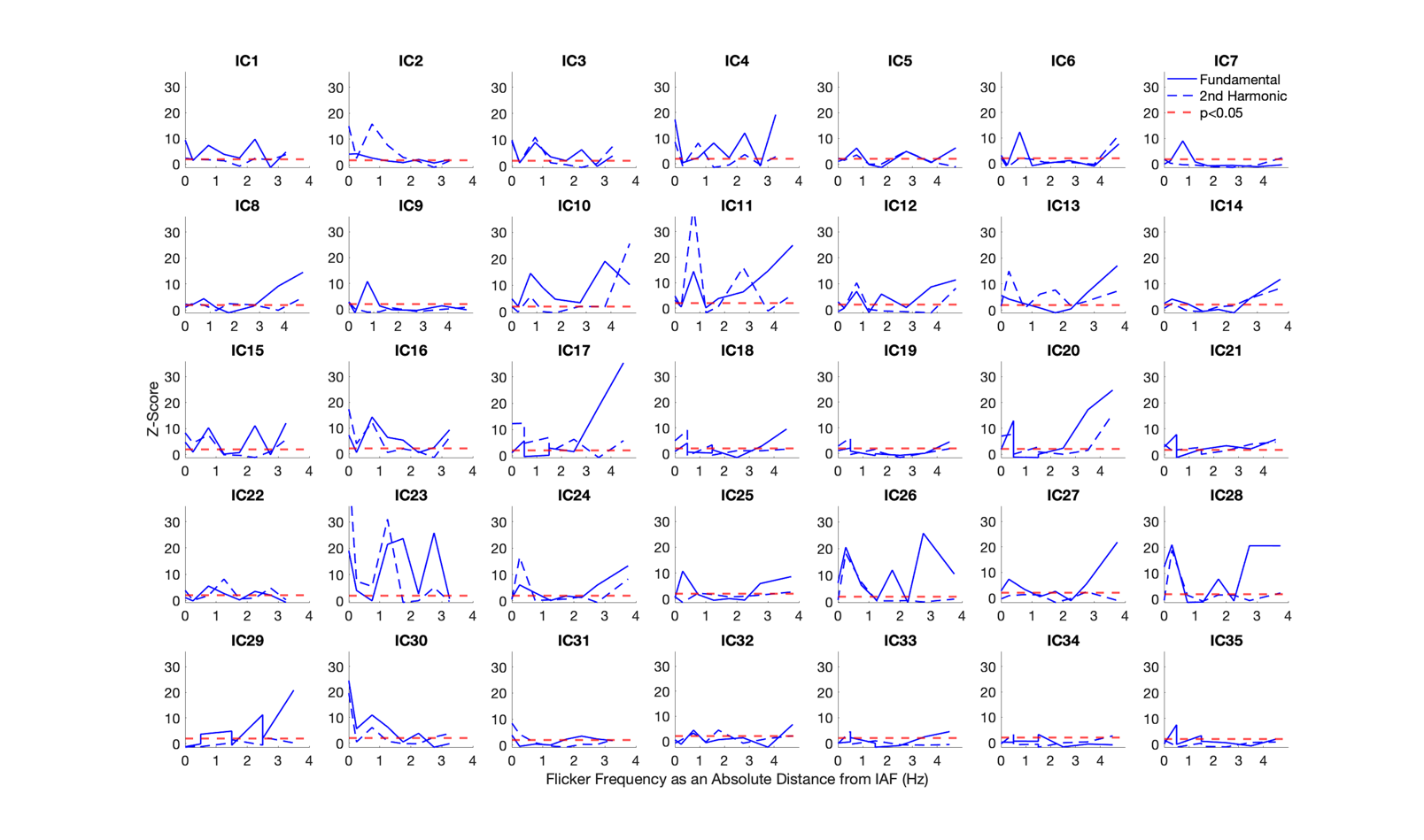


**Figure S8. The statistical significance of the evoked amplitude to flicker.** The evoked amplitude expressed as z-scores across conditions is displayed for each component, at the fundamental frequency (solid blue line) and the second harmonic (dashed blue line). A z-score threshold is illustrated with a red dashed line, corresponding to p < 0.05.
