# Supplementary figures and images for: Evoked responses to rhythmic visual stimulation vary across sources of intrinsic alpha activity in humans
Source: Sci Rep. 2022 Apr 8;12:5986. doi: 10.1038/s41598-022-09922-2 (PMC8993822; doi:10.1038/s41598-022-09922-2)

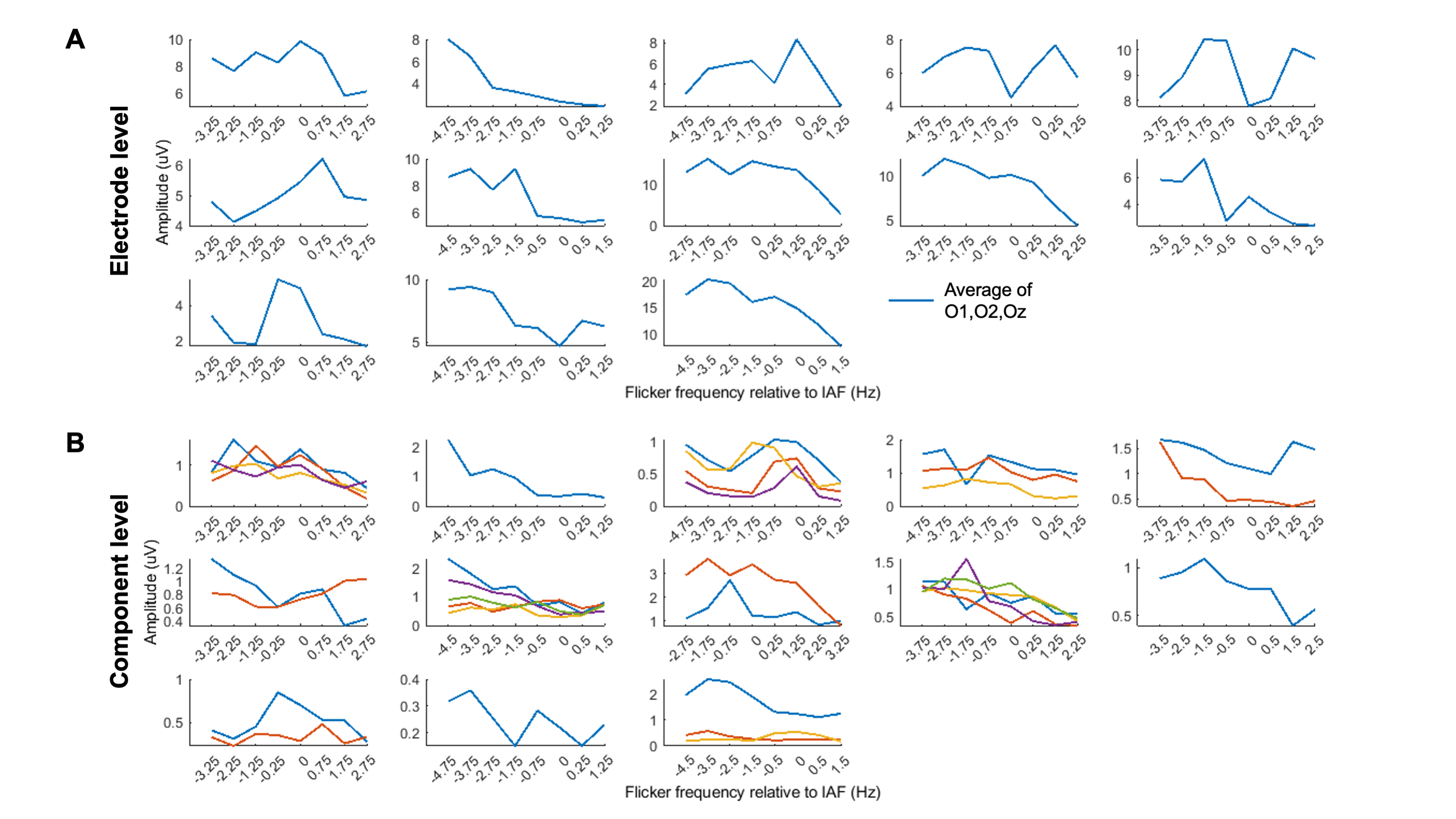

Supplement: Supplementary file 2 — Supplementary Information 2. [file 41598_2022_9922_MOESM2_ESM.tiff]

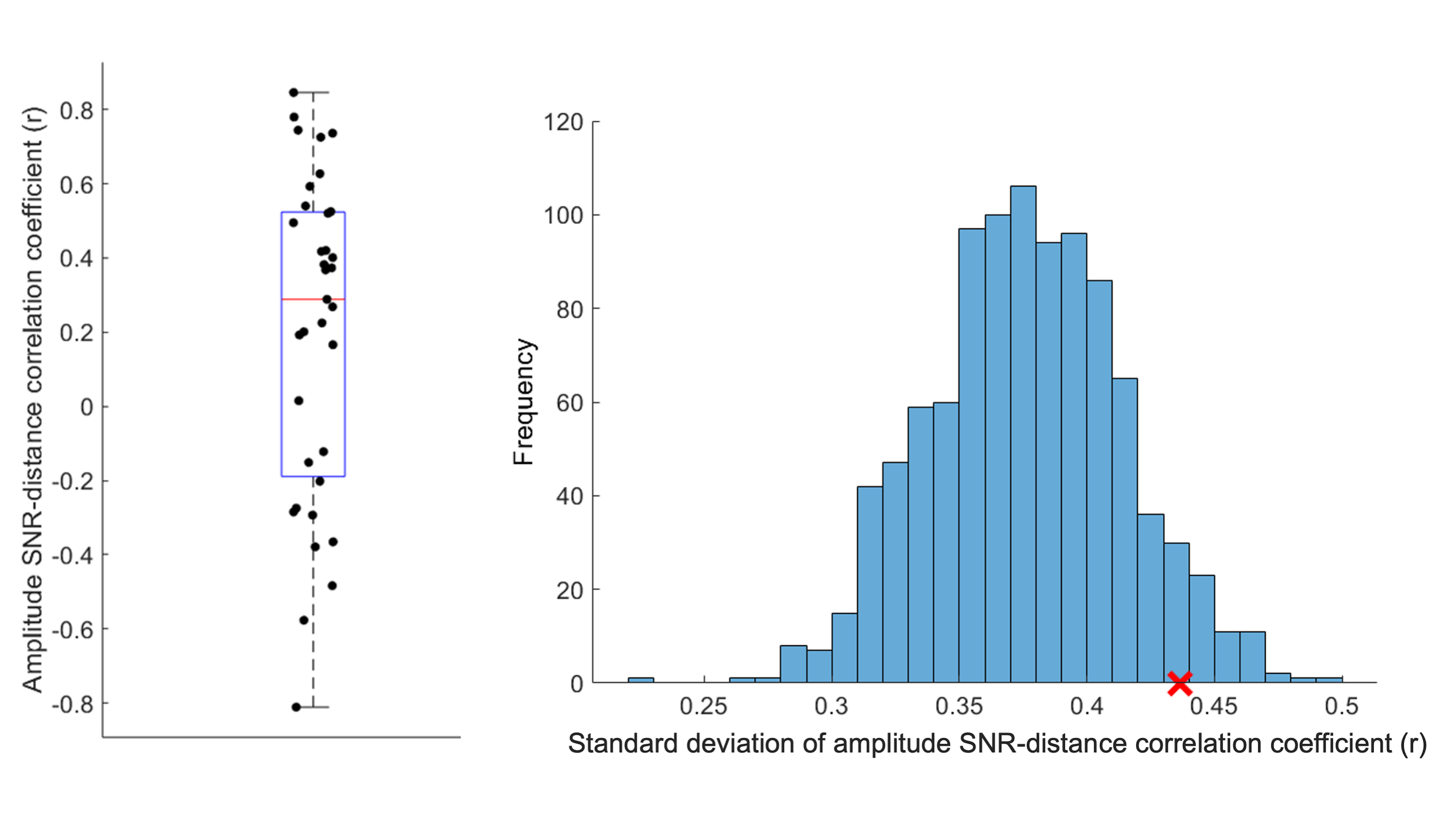

Supplement: Supplementary file 3 — Supplementary Information 3. [file 41598_2022_9922_MOESM3_ESM.tiff]

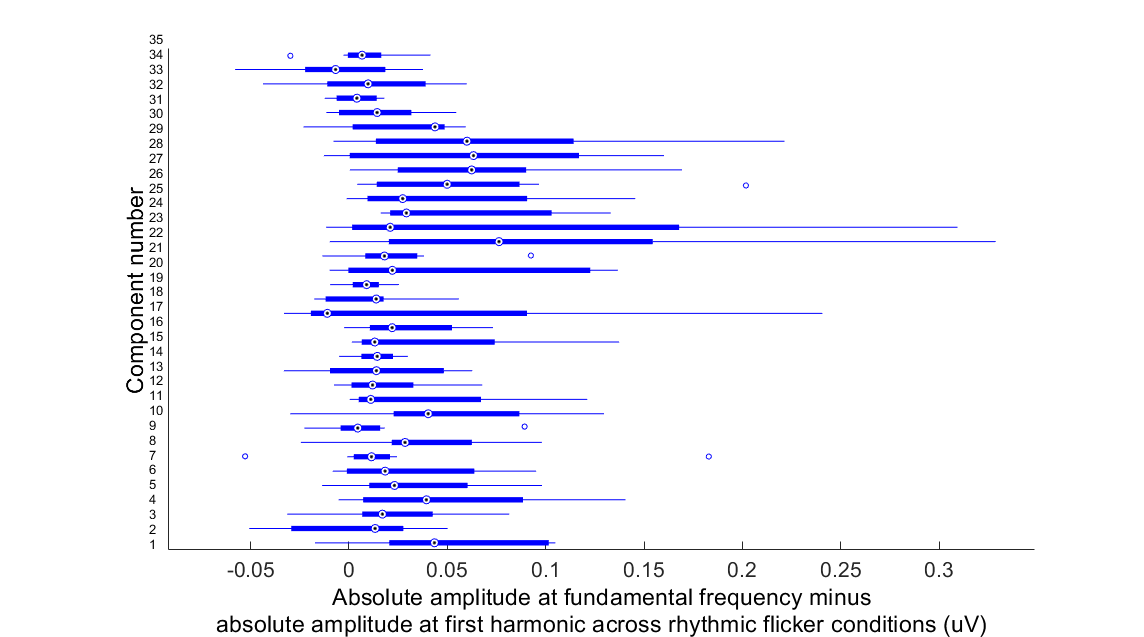

Supplement: Supplementary file 4 — Supplementary Information 4. [file 41598_2022_9922_MOESM4_ESM.tif]

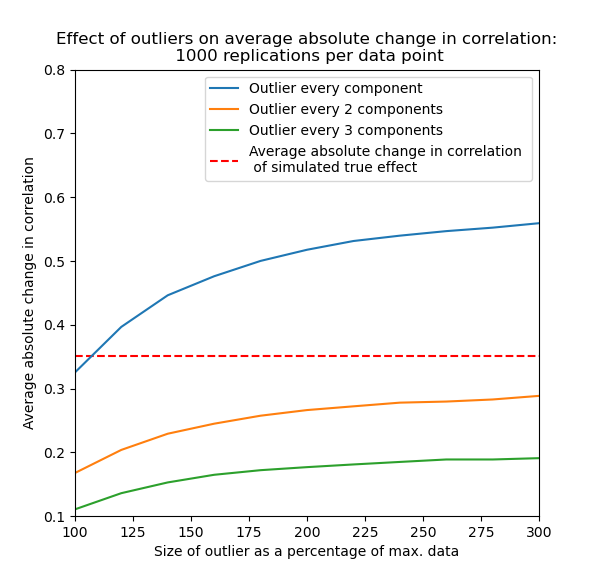

Supplement: Supplementary file 5 — Supplementary Information 5. [file 41598_2022_9922_MOESM5_ESM.tiff]

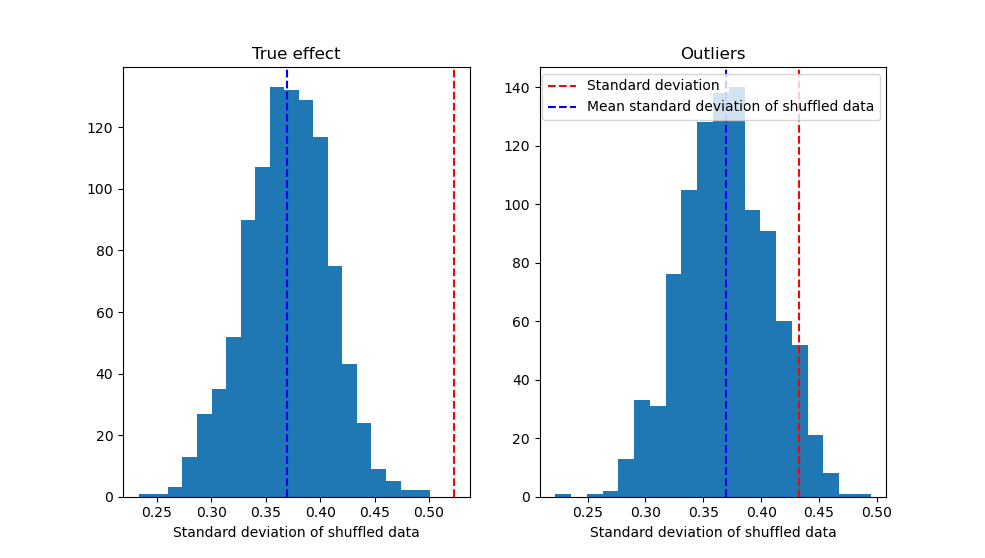

Supplement: Supplementary file 6 — Supplementary Information 6. [file 41598_2022_9922_MOESM6_ESM.tiff]

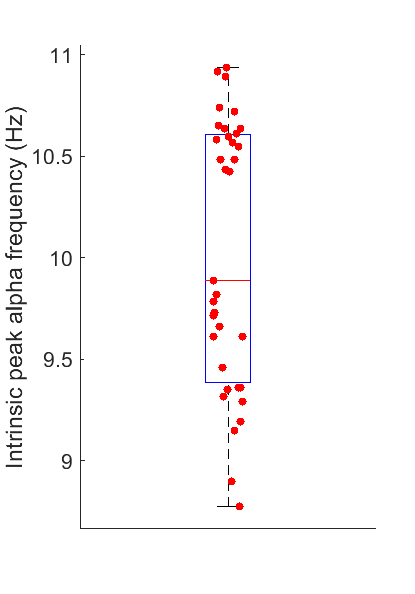

Supplement: Supplementary file 7 — Supplementary Information 7. [file 41598_2022_9922_MOESM7_ESM.tiff]

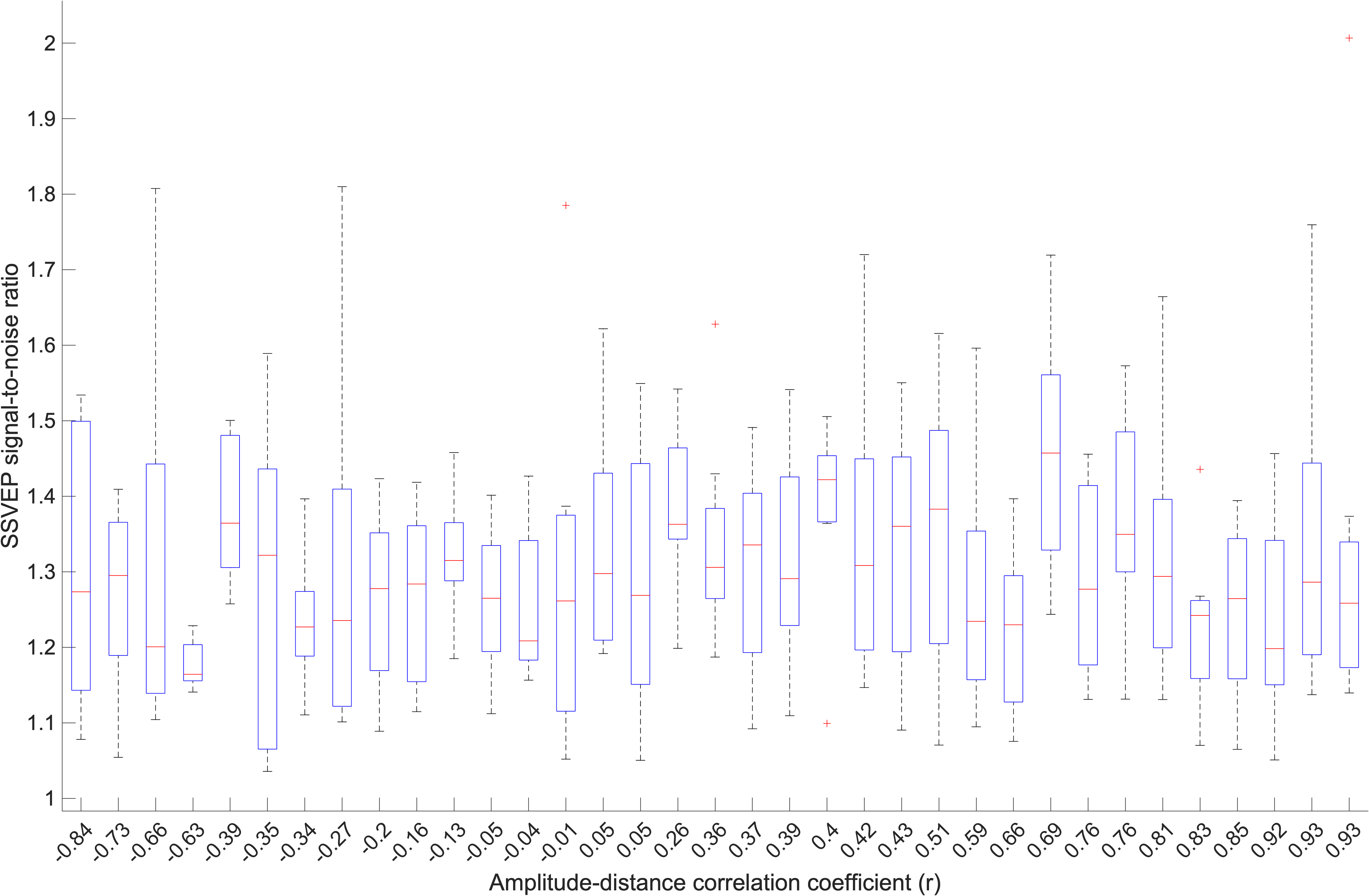

Supplement: Supplementary file 8 — Supplementary Information 8. [file 41598_2022_9922_MOESM8_ESM.tiff]
